# Interaction of LATS1 with SMAC links the MST2/Hippo pathway with apoptosis in an IAP-dependent manner

**DOI:** 10.1038/s41419-022-05147-3

**Published:** 2022-08-08

**Authors:** Lucía García-Gutiérrez, Emma Fallahi, Nourhan Aboud, Niall Quinn, David Matallanas

**Affiliations:** Systems Biology Ireland, School of Medicine, University College Dublin, Belfield, Dublin 4, Ireland

**Keywords:** Protein-protein interaction networks, Extracellular signalling molecules

## Abstract

Metastatic malignant melanoma is the deadliest skin cancer, and it is characterised by its high resistance to apoptosis. The main melanoma driving mutations are part of ERK pathway, with BRAF mutations being the most frequent ones, followed by NRAS, NF1 and MEK mutations. Increasing evidence shows that the MST2/Hippo pathway is also deregulated in melanoma. While mutations are rare, MST2/Hippo pathway core proteins expression levels are often dysregulated in melanoma. The expression of the tumour suppressor RASSF1A, a bona fide activator of the MST2 pathway, is silenced by promoter methylation in over half of melanomas and correlates with poor prognosis. Here, using mass spectrometry-based interaction proteomics we identified the Second Mitochondria-derived Activator of Caspases (SMAC) as a novel LATS1 interactor. We show that RASSF1A-dependent activation of the MST2 pathway promotes LATS1-SMAC interaction and negatively regulates the antiapoptotic signal mediated by the members of the IAP family. Moreover, proteomic experiments identified a common cluster of apoptotic regulators that bind to SMAC and LATS1. Mechanistic analysis shows that the LATS1-SMAC complex promotes XIAP ubiquitination and its subsequent degradation which ultimately results in apoptosis. Importantly, we show that the oncogenic BRAF^V600E^ mutant prevents the proapoptotic signal mediated by the LATS1-SMAC complex while treatment of melanoma cell lines with BRAF inhibitors promotes the formation of this complex, indicating that inhibition of the LATS1-SMAC might be necessary for BRAF^V600E^-driven melanoma. Finally, we show that LATS1-SMAC interaction is regulated by the SMAC mimetic Birinapant, which requires C-IAP1 inhibition and the degradation of XIAP, suggesting that the MST2 pathway is part of the mechanism of action of Birinapant. Overall, the current work shows that SMAC-dependent apoptosis is regulated by the LATS1 tumour suppressor and supports the idea that LATS1 is a signalling hub that regulates the crosstalk between the MST2 pathway, the apoptotic network and the ERK pathway.

## Introduction

Metastatic melanoma is the most aggressive type of skin cancer, and despite recent advances in the use of targeted therapy and immunotherapies, two-thirds of patients do not respond to current treatments [1–3]. Deregulation of apoptosis signals is common in this cancer type and is associated with the development of resistance to BRAF inhibitors [4–6]. For example, increased expression of negative regulator of caspases Inhibitor of Apoptosis Proteins (IAPs), has been recurrently reported in melanoma preventing anti-oncogenic signals. IAP proteins are regulated by the Second Mitochondria-derived Activator of Caspases (SMAC), which is released from the mitochondria upon a proapoptotic signal, allowing caspase activation by counteracting IAP activity. Thus, the increase of IAP expression prevents SMAC-dependent apoptosis [7, 8]. This has led to the research for novel therapeutics that can re-sensitise the cells to apoptosis signals including SMAC mimetics (SMs) [9]. While several studies have been carried out to evaluate the efficacy of SMs in melanoma cells [10], this cancer type shows poor responses SMs as single agents, and their mechanism of action is still poorly characterised [7, 9].

LATS1 is a tumour suppressor which, together with MST1/2 and YAP, forms the core signalling unit (MST1/2-LATS1/2-YAP) of the MST2/Hippo pathway (herein MST2 pathway) [11, 12]. RASSF1A, a tumour suppressor repressed by promoter methylation in more than 50% of advanced melanomas [13, 14], stimulates LATS1-dependent apoptosis both through the intrinsic and extrinsic apoptotic pathways [15]. Deregulation of LATS1 and MST2 in melanoma is also relatively common in patients [16, 17]. Thus, silencing of the proapoptotic MST2 pathway seems to be a common event in aggressive melanoma and restoration of RASSF1A expression is sufficient to induce cell death in melanoma cell lines [13]. Importantly, several lines of evidence indicate that the ERK pathway, which is commonly deregulated in melanomas due BRAF or NRAS mutations, can contribute to silencing the MST2 pathway proapoptotic signal through different mechanisms: (i) RAF1 and mutant BRAF^V600E^ can block LATS1 activity by direct binding and inhibition of MST kinases [18, 19]; (ii) activated AKT inhibits MST2 by direct phosphorylation [20]; (iii) mutant NRAS can inhibit MST2 activation, potentially through AKT activation [21]. Also, there is growing evidence supporting the importance of LATS1 in the regulation of apoptosis, including the regulation of p53, PUMA and mediation of death receptor-dependent apoptosis [15, 22, 23].

Here, we explored the role of LATS1 in the regulation of the canonical proapoptotic machinery and the potential role of this crosstalk in melanoma. We have identified SMAC as a novel interactor of LATS1 and found that this protein complex leads to XIAP degradation, one of the members of the IAP family, promoting apoptosis. Moreover, this interaction is dependent on RASSF1A expression and another member of the IAP family C-IAP1 negatively regulates it. Finally, we show that the restoration of LATS1 proapoptotic signalling in combination with SMs can restore the sensitivity of melanoma cells to apoptosis.

## Results

### LATS1 induces apoptosis in a SMAC-dependent manner

**Fig. 1:**
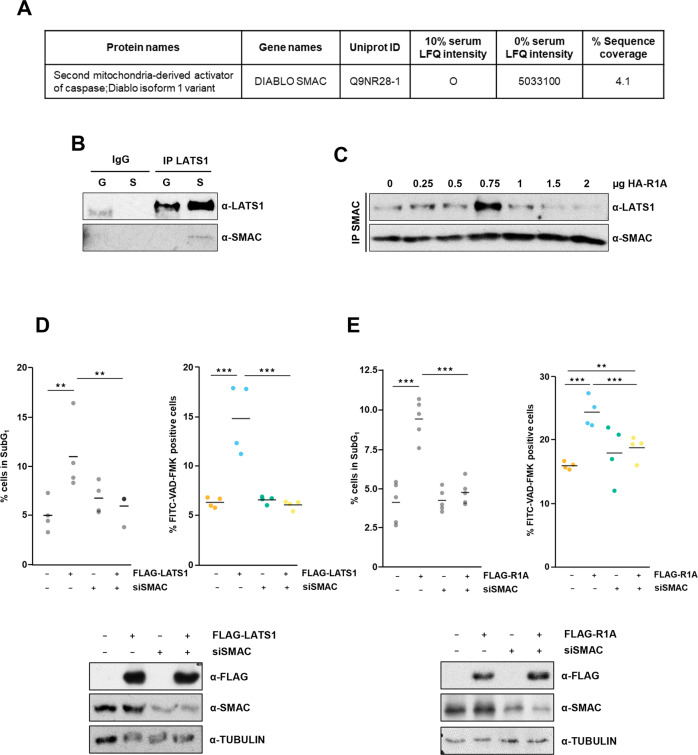
LATS1-mediated cell death is dependent on SMAC levels. **A** HEK293 cells transfected with FLAG-LATS1 were lysed and LATS1 interactome was identified using mass spectrometry. The table shows the results for SMAC protein in LATS1 from MaxQuant search. LFQ (label-free quantitation) intensity is shown for cells cultured in either complete media (10% serum-supplemented media) or serum deprived (0% serum-supplemented media). **B** Endogenous interaction of LATS1 with SMAC in HeLa cells assessed by co-immunoprecipitation upon proapoptotic serum-deprived conditions. Levels of immunoprecipitated LATS1 and SMAC are shown. Normal IgG was used as negative control. **C** Endogenous interaction of LATS1 with SMAC in MCF7 cells upon increasing RASSF1A (R1A) expression and serum deprivation. Levels of immunoprecipitated SMAC and LATS1 are shown. **D** Left: percentage of HeLa cells in subG_1_ phase upon LATS1 overexpression and SMAC downregulation in serum-deprived conditions determined by flow cytometry after PI staining (*n* = 3). Right: Percentage of HeLa cells showing Caspase 3 activation upon LATS1 overexpression and SMAC downregulation in serum-deprived conditions determined by flow cytometry after FITC-VAD-FMK staining (*n* = 4). Lower panel: FLAG-LATS1 overexpression and SMAC downregulation measured by western blot. TUBULIN was used as loading control. **E** Left: Percentage of MCF7 cells in subG_1_ phase upon RASSF1A (R1A) overexpression and SMAC downregulation in serum-deprived conditions determined by flow cytometry after PI staining (*n* = 4). Right: Percentage of MCF7 cells showing Caspase 3 activation upon RASSF1A (R1A) overexpression and SMAC downregulation in serum-deprived conditions determined by flow cytometry after FITC-VAD-FMK staining (*n* = 4). Lower panel: FLAG-RASSF1A (FLAG-R1A) overexpression and SMAC downregulation measured by western blot. TUBULIN was used as loading control. Statistical analysis: *t* test *p* ≤ 0.1*; *p* ≤ 0.05**; *p* ≤ 0.01***.

During the last decade, using affinity purification proteomics mass spectrometry we have identified MDM2-TP53, YAP1-TP73 as effectors and IQGAP as regulator of the LATS1 proapoptotic signal [24–26]. However, we still lack a complete picture of the LATS1 proapoptotic network. We mined two sets of interaction proteomics experiments to identify novel LATS1 interactors that are part of LATS1 proapoptotic machinery [27]. These analyses confirmed the enrichment of apoptotic proteins in the LATS1 interactome, when HEK293 cells were serum-deprived. Intriguingly, we identified SMAC as a putative LATS1 interactor upon proapoptotic conditions (Fig. 1A). LATS1-SMAC interaction was validated in serum-deprived HeLa cells, a cell line that expresses RASSF1A (Fig. 1B). In agreement, re-expression of RASSF1A in MCF7, a cell line where RASSF1A expression is lost due to DNA methylation, promotes LATS1-SMAC interaction indicating that this scaffold is a regulator of this complex (Fig. 1C) [15, 25]. We tested whether SMAC mediates RASSF1A-LATS1-dependent apoptosis in these cell lines. The data show that LATS1-mediated cell death is rescued by downregulation of SMAC in HeLa cells (Fig. 1D), and SMAC knockdown impaired RASSF1A-mediated apoptosis in MCF7 cells (Fig. 1E), confirming that SMAC mediates RASSF1A-LATS1 dependent apoptosis.

### Expression of RASSF1A promotes apoptosis and LATS1 activation in melanoma cells

**Fig. 2:**
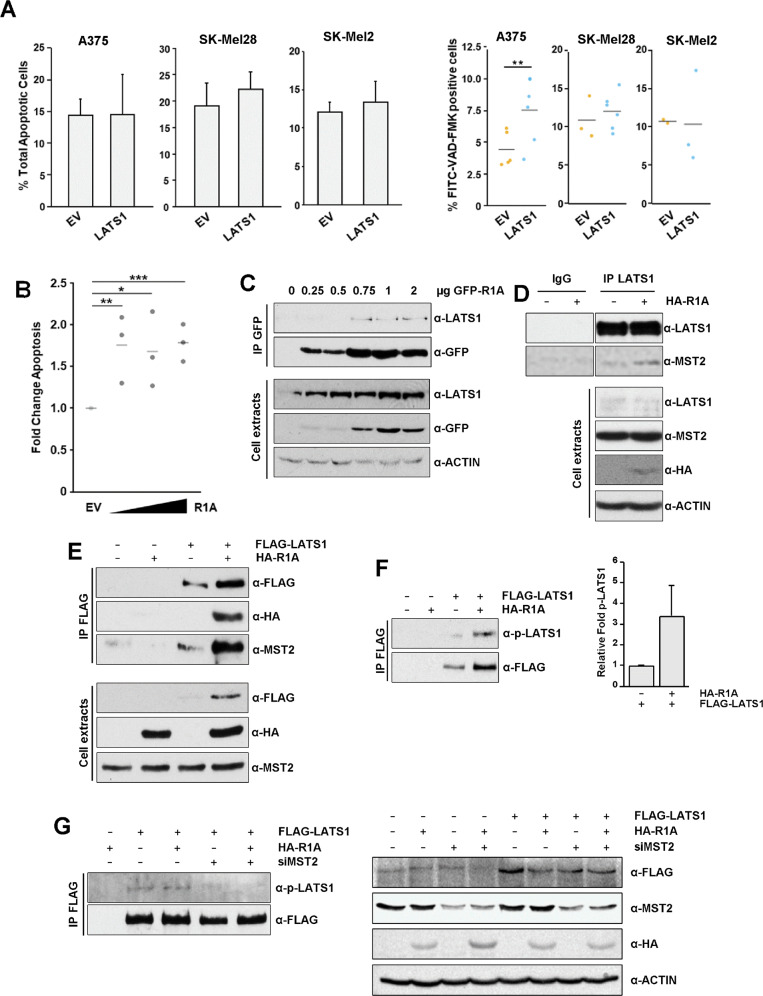
RASSF1A promotes apoptosis and LATS1 activation and stabilisation in BRAF melanoma cells. **A** Left: percentage of apoptotic A375, SK-Mel28 and SK-Mel2 melanoma cells upon LATS1 overexpression in serum-deprived conditions measured by flow cytometry after YoPro-PI staining (*n* = 2). Right: Percentage of A375, SK-Mel28 and SKMel2 cells showing Caspase 3 activation upon LATS1 overexpression in serum-deprived conditions determined by flow cytometry after FITC-VAD-FMK staining. Statistical analysis: *t* test *p* ≤ 0.05**. **B** Apoptosis induction of A375 melanoma cells upon restoration of RASSF1A expression. Cells were transfected with increasing amounts of GFP-RASSF1A (R1A) and apoptosis measured after 24 h by YoPro-PI staining. Data shows fold change of apoptosis from 3 independent experiments and bars represent averages. Statistical analysis: *t* test *p* ≤ 0.1*; *p* ≤ 0.05**; *p* ≤ 0.01***. **C** LATS1-RASSF1A interaction upon RASSF1A re-expression in A375 melanoma cells. GFP-RASSF1A was immunoprecipitated and LATS1 co-immunoprecipitated levels are shown (upper panel). Total levels of LATS1 and transfected GFP-RASSF1A (lower panel). **D** MST2-LATS1 interaction in A375 melanoma cells upon RASSF1A re-expression. Endogenous LATS1 was immunoprecipitated. Levels of immunoprecipitated LATS1 and co-immunoprecipitated MST2 are shown (upper panel). Normal rabbit IgG was used as negative control. Lower panel shows total LATS1 and MST2 levels. Transfected RASSF1A was detected using an anti-HA probe. Actin levels were used as loading control. **E** Interaction between FLAG-LATS1 and endogenous MST2 in A375 melanoma cells transfected with either HA-RASSF1A or the corresponding empty vector. Levels of immunoprecipitated FLAG-LATS1 and MST2 and RASSF1A binding are shown (upper panel). Total lysates of A375 melanoma cells transfected with FLAG-LATS1 and HA-RASSF1A as indicated or the corresponding empty vectors. Expression levels of transfected FLAG-LATS1, HA-RASSF1A and endogenous MST2 are shown (lower panel). **F** LATS1 phosphorylation levels upon RASSF1A re-expression in A375 melanoma cells. FLAG-LATS1 was immunoprecipitated using anti-FLAG beads in presence of HA-RASSF1A or the corresponding empty vector and levels of phosphorylated LATS1 were assayed by western blot (left panel). Right panel shows densitometric quantification of LATS1 phosphorylation upon RASSF1A expression in A375 cells corresponding to the left panel. Data represent ±S.D. of two independent experiments. **G** Left panel: LATS1 phosphorylation levels upon RASSF1A re-expression in A375 melanoma cells and concomitant knockdown of MST2 levels by siRNA transfection. FLAG-LATS1 was immunoprecipitated using anti-FLAG beads and levels of phosphorylated LATS1 were assayed by western blot. Right panel: Total levels of transfected FLAG-LATS1 and HA-RASSF1A (HA-R1A) and endogenous MST2 corresponding to left panel. ACTIN was used as loading control.

Given the known role of IAP-SMAC deregulation in skin cancers and the lack of characterisation of LATS1-dependent apoptosis in melanoma we wanted to study the possible role of the novel LATS1-SMAC interaction in this cancer type. As a first step, LATS1 was overexpressed in three different melanoma cell lines. Unlike what we observed in HeLa cells, all of them were resistant to LATS1-mediated apoptosis (Fig. 2A). Interestingly, LATS1 induced a significant increase in Caspase 3 activation in A375 melanoma cells (unlike the other two melanoma cell lines assayed) which was not enough to promote cell death (Fig. 2A). Since RASSF1A expression is lost in these cell lines [19, 28, 29], we postulated that the lack of expression of RASSF1A could impair LATS1 proapoptotic signalling in melanoma, explaining the resistance to LATS1 overexpression observed in these cell lines. Hence, we studied the effect that RASSF1A re-expression had in the regulation of MST2-LATS1 interaction in A375 cells. First, we saw that RASSF1A re-expression induced apoptosis as previously reported (Fig. 2B). Next, we observed that the induction of apoptosis correlated with LATS1-RASSF1A interaction (Fig. 2C) and seemed to play a role in LATS1 protein stability (Fig. 2C). Expression of RASSF1A also led to an increase in LATS1-MST2 interaction (Fig. 2D, E) that was accompanied by the activation of LATS1, as shown by LATS1 phosphorylation levels (Fig. 2F). Interestingly, LATS1 phosphorylation was completely abolished by MST2 downregulation (Fig. 2G), showing that RASSF1A requires MST2 to promote LATS1 activation. These experiments indicate that the proapoptotic LATS1 signalling is compromised in this cell line, at least in part, due to lack of expression of RASSF1A.

### LATS1 interacts with SMAC in BRAF^V600E^-driven A375 melanoma cells in a RASSF1A/MST2-dependent manner

Our previous results suggested that RASSF1A was regulating LATS1-SMAC interaction, and that RASSF1A regulates LATS1 signalling in melanoma. Hence, we further studied the interaction of LATS1 and SMAC in A375 cells to characterise the dynamics of this protein complex formation and its biochemical function.

**Fig. 3:**
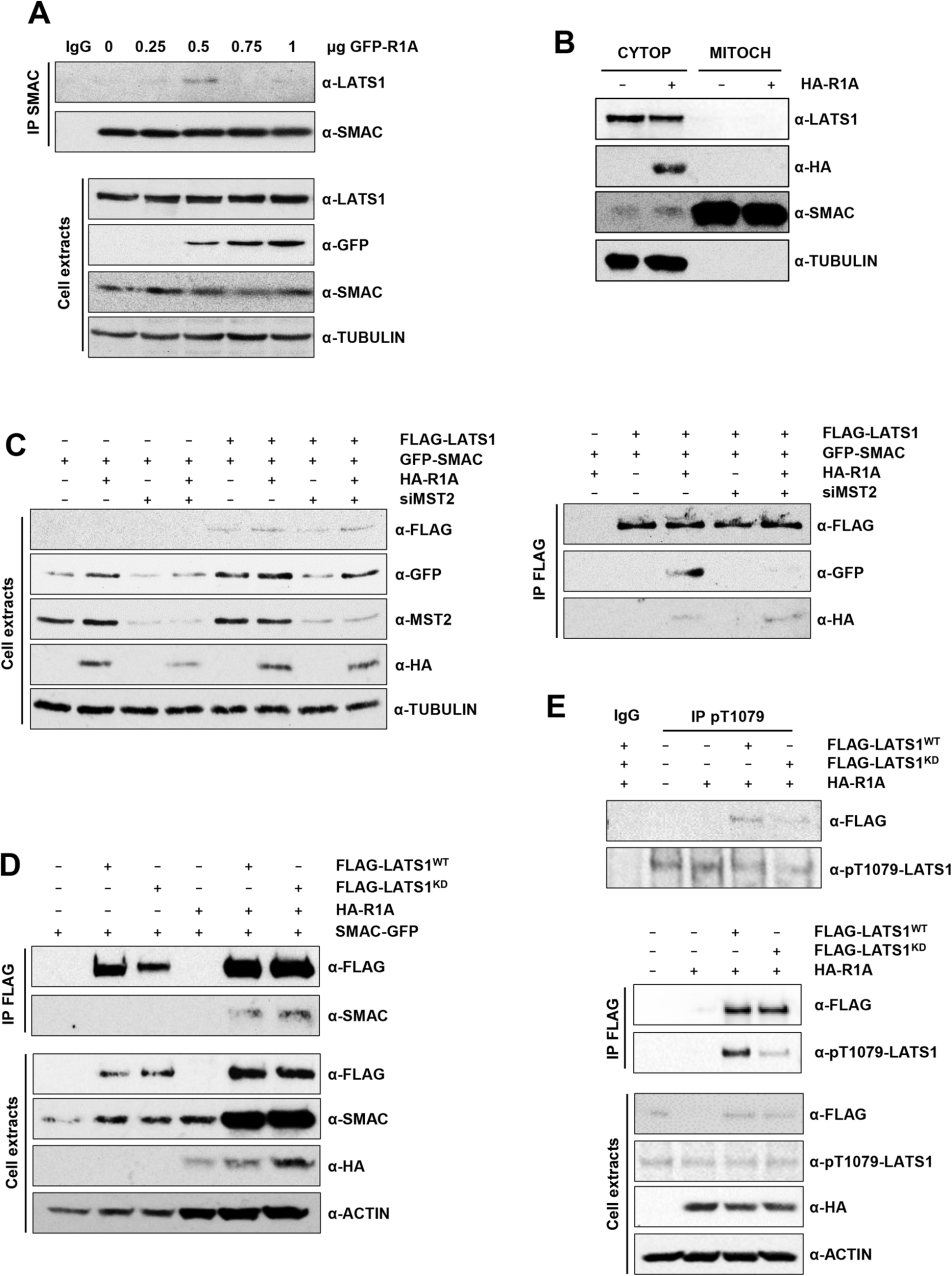
Re-expression of RASSF1A promotes LATS1-SMAC interaction in A375 melanoma cells. **A** Interaction of endogenous SMAC with LATS1 in A375 cells assessed by co-immunoprecipitation assay followed by western blot analysis upon increasing amounts of GFP-RASSF1A in serum deprived conditions. Levels of immunoprecipitated SMAC and co-immunoprecipitated LATS1 are shown (upper panel). Total LATS1, SMAC and transfected GFP-RASSF1A protein levels (lower panel). Tubulin was used as loading control. **B** Cytoplasmic-Mitochondrial fractionation in A375 cells transfected with HA-RASSF1A upon serum deprivation. Total lysates corresponding to cytoplasmic and mitochondrial fractions were assessed by western blot. LATS1, SMAC, Tubulin and transfected RASSF1A-HA levels are shown. **C** Exogenous LATS1-SMAC interaction in A375 cells upon MST2 downregulation. Levels of immunoprecipitated FLAG-LATS1 and co-immunoprecipitated GFP-SMAC and HA-RASSF1A are shown (right panel). Total protein levels of A375 transfected with FLAG-LATS1, GFP-SMAC and HA-RASSF1A and MST2 downregulation assayed by western blot (left panel). Tubulin was used as loading control. **D** Interaction between SMAC and LATS1 wild-type (LATS1^WT^) or LATS1 kinase dead mutant (LATS1^KD^) upon RASSF1A re-expression and serum deprivation. Levels of immunoprecipitated FLAG-LATS1 and co-immunoprecipitated GFP-SMAC are shown (upper panel). Total protein levels of A375 transfected with FLAG-LATS1^WT^ or FLAG-LATS1^KD^, GFP-SMAC and HA-RASSF1A assayed by western blot (lower panel). Actin was used as loading control. **E** Phosphorylation state of LATS1^WT^ and LATS1^KD^ at the T1079 upon HA-RASSF1A expression in A375 cells. Upper panel: phosphorylated LATS1 was immunoprecipitated using an anti-phospho-T1079-LATS1 specific antibody and presence of exogenous LATS1, either wild-type (WT) or kinase dead (KD) was detected with anti-FLAG. Normal rabbit IgG was used as negative control. Middle panel: phosphorylated levels of LATS1^WT^ and LATS1^KD^ at the T1079 measured by western blot after FLAG-LATS1 immunoprecipitation. Lower panel: Total levels of transfected FLAG-LATS1 and HA-RASSF1A (HA-R1A) and phospho-LATS1 (pT1079-LATS1). ACTIN was used as loading control.

First, we assayed LATS1 interaction with SMAC in A375 cells upon increasing amounts of RASSF1A. We found that SMAC binds to LATS1 in a RASSF1A-dependent fashion, in agreement with the scaffolding nature of this protein (Fig. 3A and Fig. S1A). We also confirmed that the LATS1-SMAC endogenous interaction is regulated in a RASSF1A-dependent manner in another BRAF-driven melanoma cells SK-Mel239 and in the NRAS-driven melanoma cells SK-Mel2 (Fig. S1B, C). Second, we focussed on the identification of the cellular compartment where LATS1-SMAC interaction takes place to better understand its biological function. SMAC is a mitochondrial protein released to the cytoplasm upon an apoptotic signal, whereas LATS1 subcellular localisation is less well studied. We isolated cytoplasmic and mitochondrial fractions from A375 cells transfected with RASSF1A and assayed protein localisation by western blot. As expected, we saw most of SMAC localised in the mitochondria and re-expression of RASSF1A causes a small increase of cytoplasmic SMAC. Conversely, no mitochondrial localisation of LATS1 or RASSF1A was observed (Fig. 3B). Thus, the data indicates that SMAC interacts with LATS1 upon its release to the cytoplasm.

Next, we studied whether the RASSF1A-dependent regulation of LATS1-SMAC interaction requires MST2. To do so, we knocked down MST2 using siRNAs and transfected RASSF1A in A375 cells and found that the RASSF1A-dependent increase of LATS1-SMAC interaction is prevented by downregulating MST2 expression (Fig. 3C). This confirms that MST2 may be needed for the formation of this complex. Of note, knocking down MST2 did not affect LATS1 interaction with RASSF1A (Fig. 3C). Interestingly, a kinase-dead LATS1 mutant (LATS1^KD^) interacted with SMAC to a similar extent as LATS1^WT^ did in a RASSF1A-dependent manner (Fig. 3D). This mutant is much less efficiently phosphorylated at the T1079 (Fig. 3E), suggesting that MST2 mediated phosphorylation of LATS1 is not needed for the interaction between LATS1 and SMAC to happen. LATS1^KD^ acts as a dominant inhibitory mutant of endogenous LATS1 [30], indicating that LATS1 kinase activity is not necessary for its interaction with SMAC. These results suggest that MST2 is essential to mediate RASSF1A regulation of LATS1 in this cell line. Altogether, these experiments clearly showed that the core proteins of the proapoptotic MST2 pathway regulate LATS1-SMAC interaction in melanoma cells.

### Identification of potential regulators of LATS1-SMAC complex formation and dynamics using quantitative mass spectrometry

To better understand the dynamics of LATS1-SMAC interaction and to find potential regulators of this complex, we used two different approaches to identify the LATS1 and SMAC interactomes by mass spectrometry in the presence or absence of RASSF1A (Fig. S2A).

**Fig. 4:**
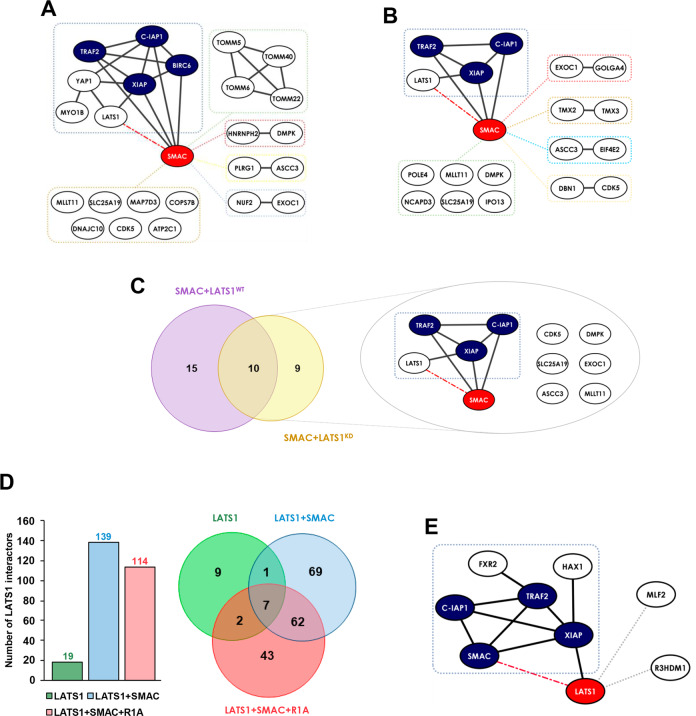
Pathway reconstruction of SMAC and LATS1 dynamic interactomes. **A** Visualisation of the GFP-SMAC interactors regulated by FLAG-LATS1^WT^ in HeLa cells obtained by AP-MS after pathway reconstruction analysis (STRING database). Black edges represent reported interactions. Dash lines represent new interactions. Red node represents the bait (SMAC). Blue nodes represent the selected core components of the LATS1-SMAC complex. **B** Visualisation of the GFP-SMAC interactors regulated by FLAG-LATS1^KD^ in HeLa cells obtained by AP-MS after pathway reconstruction analysis (STRING database). Black edges represent reported interactions. Dash lines represent new interactions. Red node represents the bait (SMAC). Blue nodes represent the selected core components of the LATS1-SMAC complex. **C** Venn diagram representing the SMAC interactors modulated by LATS1 indicating the amount of them shared by FLAG-LATS1^WT^ and FLAG-LATS1^KD^ (left panel). Visualisation of the cluster of SMAC interactors regulated by both LATS1^WT^ and LATS1^KD^ after pathway reconstruction analysis (STRING database). **D** Graph representing the number of specific proteins interacting with FLAG-LATS1 after AP-MS in A375 cells expressing either LATS1 alone or in combination with GFP-SMAC or GFP-SMAC and FLAG-RASSF1A as indicated (left panel). Venn diagram representing the number of LATS1 interactors which are either specific or shared among the different conditions (right panel). **E** Visualisation of the proteins interacting with FLAG-LATS1 differentially regulated by SMAC in A375 cells upon RASSF1A re-expression (i.e. fold ≤ 0.66 or fold ≥ 1.5 of LATS1 + SMAC vs LATS1 + SMAC + RASSF1A). Pathway reconstruction analysis is represented (STRING database). Red node represents the bait (LATS1). Blue nodes represent the selected core components of the LATS1-SMAC complex. Black edges represent reported interactions. Dash lines represent new interactions.

First, we used HEK293 cells transiently transfected with a GFP-tagged SMAC and either a wild-type or a kinase-dead FLAG-tagged LATS1 construct (LATS1^WT^-FLAG or LATS1^KD^-FLAG, respectively). After serum deprivation, GFP-SMAC was immunoprecipitated and subjected to mass spectrometry analysis. This screening confirmed that both LATS1^WT^ and LATS1^KD^ interact with SMAC (Table S1), in agreement with our previous results (Fig. 3D). While the SMAC interactome yielded a total of 381 potential specific interactors, co-transfection of either FLAG-LATS1^WT^ or FLAG-LATS1^KD^ constructs led to a subset of 25 and 19 differentially regulated SMAC interactors respectively (Table S1). Of the 44 proteins that are regulated by LATS1 expression, 10 were shared between the two conditions, suggesting that LATS1 regulates SMAC interactome in a kinase-dependent and independent fashion. We performed pathway reconstruction analysis to identify clusters of proteins already curated in the data bases (Fig. 4A, B). The analysis showed that the SMAC interactors regulated by LATS1^WT^ overexpression were grouped in five clusters, which include the well-known interactors of the IAP family including XIAP, C-IAP1 and BIRC6, and TRAF2, and other four modules, including TOMMs, which have not been reported to interact with SMAC before (Fig. 4A). Importantly, LATS1^KD^ expression differentially regulates other 5 clusters, including the one that consisted of XIAP, C-IAP1, and TRAF2, while the interaction with YAP and BIRC6 was lost (Fig. 4B). Thus, the group of SMAC interactors that included LATS1 was shared between both conditions (WT and KD) and consists of XIAP, C-IAP1, and TRAF2, suggesting that this could be the core components of this protein complex (Fig. 4C).

The second affinity purification-mass spectrometry (AP-MS) screen was performed in A375 cells to understand how SMAC levels regulate LATS1 interactome in melanoma cells. To do so, we identified the interactome of LATS1 by mass spectrometry using a FLAG-LATS1 construct co-transfected with GFP-SMAC alone or in combination with RASSF1A. We obtained a list of 19 specific interactors of LATS1 in basal conditions, while this number increased to 139 upon SMAC expression (Fig. 4D and table S2), confirming that SMAC can regulate LATS1 interactome. Co-expression of RASSF1A and SMAC yielded a total of 114 LATS1-specific interactors (Fig. 4D and Table S2). Reassuringly, SMAC was found within a group of 62 LATS1 interactors shared by conditions of SMAC overexpression, alone or in combination with RASSF1A. Additionally, we identified a list of 127 LATS1 interactors differentially regulated by SMAC overexpression that were used to perform cluster enrichment analysis (Fig. S2B and Table S2). Interestingly, this analysis showed that LATS1 is directly connected to the cluster of proteins composed of IAPs, SMAC, TRAF1/2 and TAB1/3 at the level of XIAP, all of them enriched upon SMAC expression. This confirms the close relation of LATS1 and SMAC and suggests a series of proteins that may be involved in the regulation of this complex formation. Next, we obtained a list of 33 proteins differentially regulated by RASSF1A and SMAC co-expression of which 28 were enriched upon RASSF1A expression, including SMAC, XIAP, C-IAP1 and TRAF2 (Table S2). Cluster enrichment analysis identified four additional proteins clusters, of which 2 of them were not linked to LATS1 before (Fig. S2C). Importantly, RASSF1A expression increased LATS1 interaction with SMAC, XIAP, C-IAP1 and TRAF2 proteins (Fig. 4E). Hence, SMAC and LATS1 interactome screening show a common cluster of intrinsic apoptosis regulators that are dynamically regulated by proapoptotic conditions.

### LATS1 interacts with XIAP and promotes its ubiquitination

**Fig. 5:**
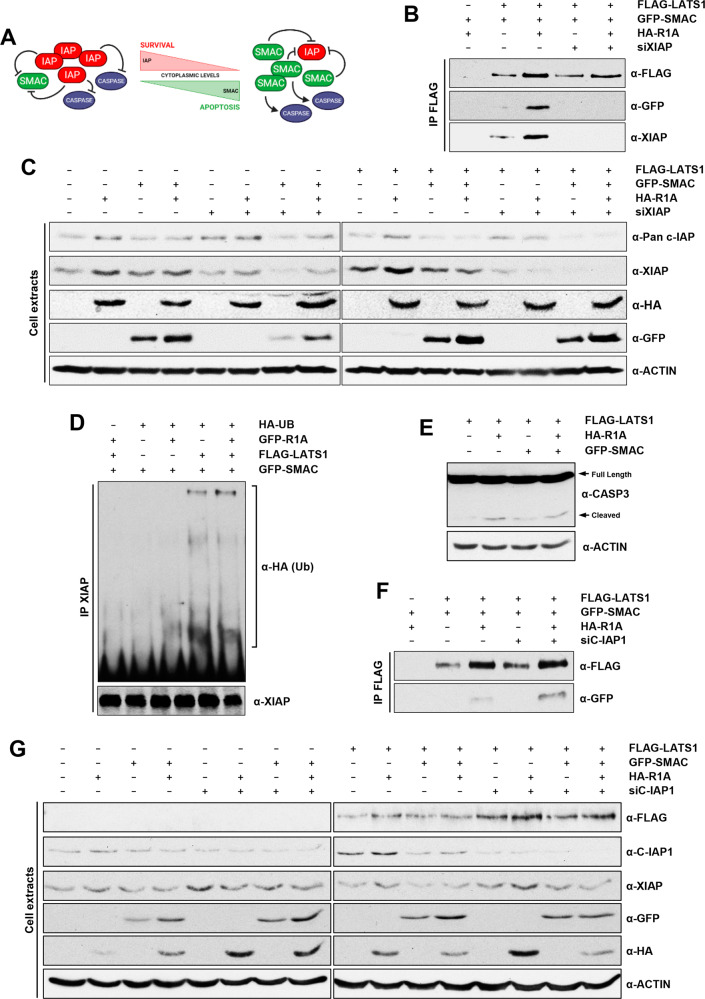
LATS1-SMAC interaction correlates with XIAP degradation and caspase cleavage. **A** Simplified scheme depicting regulation dynamics between IAPs, SMAC and caspases upon survival or apoptotic conditions. **B** Exogenous LATS1-SMAC interaction in A375 cells transfected with FLAG-LATS1 and GFP-SMAC assessed by co-immunoprecipitation assay followed by western blot analysis upon knocking down XIAP levels using specific siRNA. Cells were also transfected with HA-RASSF1A or the corresponding empty vector and serum-deprived overnight. FLAG-LATS1 was immunoprecipitated using anti-FLAG beads. Levels of immunoprecipitated FLAG-LATS1, GFP-SMAC and XIAP are shown. **C** Total lysates of A375 cells transfected with the indicated plasmids and siRNAs or their respective controls corresponding to B) assayed by western blot. Levels of transfected FLAG-LATS1, GFP-SMAC and HA-RASSF1A and endogenous C-IAP and XIAP are shown. Actin was used as loading control. Samples run in two separate gels which were transferred together to the same membrane to compare protein levels between two different gels. **D** XIAP ubiquitination approach measured by western blot. A375 cells were transfected with FLAG-LATS1, GFP-SMAC and GFP-RASSF1A as indicated and endogenous XIAP was immunoprecipitated after serum deprivation. HA-Ub was co-transfected and XIAP ubiquitination was measured by detecting HA. Immunoprecipitated XIAP levels are shown. **E** Caspase 3 cleavage of A375 cells transfected with FLAG-LATS1, HA-RASSF1A and GFP-SMAC as indicated upon serum deprivation. Caspase 3 cleavage was measured by western blot. Actin was used as loading control. **F** Exogenous LATS1-SMAC interaction in A375 cells transfected with FLAG-LATS1 and GFP-SMAC assessed by co-immunoprecipitation assay followed by western blot analysis upon knocking down C-IAP1 levels using specific siRNA. Cells were also transfected with HA-RASSF1A or the corresponding empty vector and serum-deprived overnight. FLAG-LATS1 was immunoprecipitated using an anti-FLAG beads. Levels of immunoprecipitated FLAG-LATS1 and GFP-SMAC are shown. **G** Total lysates of A375 cells transfected with the indicated plasmids and siRNAs or their respective controls corresponding to F) assayed by western blot. Levels of transfected FLAG-LATS1, GFP-SMAC, HA-RASSF1A and endogenous C-IAP1 and XIAP are shown. Actin was used as loading control. Samples run in two separate gels which were transferred together to the same membrane to compare protein levels between two different gels.

IAP proteins and SMAC have opposite roles in the regulation of apoptosis and reciprocally regulate each other (Fig. 5A) [31]. Thus, we were particularly interested in studying the role that XIAP and C-IAP1 have on the LATS1-SMAC complex. First, we validated the interaction of LATS1 with XIAP and found that RASSF1A enhanced this interaction (Fig. 5B). Also, XIAP downregulation abolished the LATS1-SMAC interaction and RASSF1A did not restore it (Fig. 5B). Total protein extracts show that RASSF1A stabilises XIAP levels and SMAC overexpression does not affect it (Fig. 5C). However, when SMAC and LATS1 are co-transfected, XIAP levels are reduced. Thus, it is possible that the LATS1-SMAC protein complex is regulating XIAP stability. This correlates with the fact that SMAC overexpression itself does not promote cell death, but it needs a further proapoptotic stimulus instead, i.e. LATS1 enforced expression. To test this, we checked for the ability of LATS1 to promote XIAP ubiquitination in the presence of high levels of SMAC. We found that SMAC overexpression alone did not promote XIAP ubiquitination, while it was slightly induced when in combination with RASSF1A restoration. Interestingly, co-expression of LATS1 alone or together with RASSF1A substantially enhanced XIAP ubiquitination (Fig. 5D). In fact, LATS1 alone yielded similar XIAP ubiquitination as when co-expressed with RASSF1A, suggesting that LATS1 plays an important role in this process. Finally, we checked for caspase 3 activation in A375 cells and found that LATS1 alone did not promote caspase 3 cleavage, in line with the lack of apoptosis induction shown in Fig. 1. However, co-expression of RASSF1A or SMAC did increase caspase 3 cleavage (Fig. 5E). As our interaction proteomics data indicated that C-IAP1 can interact with LATS1, we checked for C-IAP1 interaction with LATS1 by co-immunoprecipitation, but we could not detect it. However, we found that downregulation of C-IAP1 enhanced LATS1-SMAC interaction in the presence of RASSF1A (Fig. 5F), suggesting that C-IAP1 may prevent the LATS1-SMAC interaction and subsequent XIAP degradation. In fact, LATS1 enhances XIAP downregulation upon concomitant SMAC overexpression and C-IAP1 knockdown (Fig. 5G). Altogether, this data shows that LATS1 forms a complex with SMAC and XIAP that promotes XIAP ubiquitination, which is enhanced by RASSF1A expression and negatively regulated by C-IAP1.

### BRAF inhibition activates the LATS1 proapoptotic signalling, and it is enhanced by RASSF1A

**Fig. 6:**
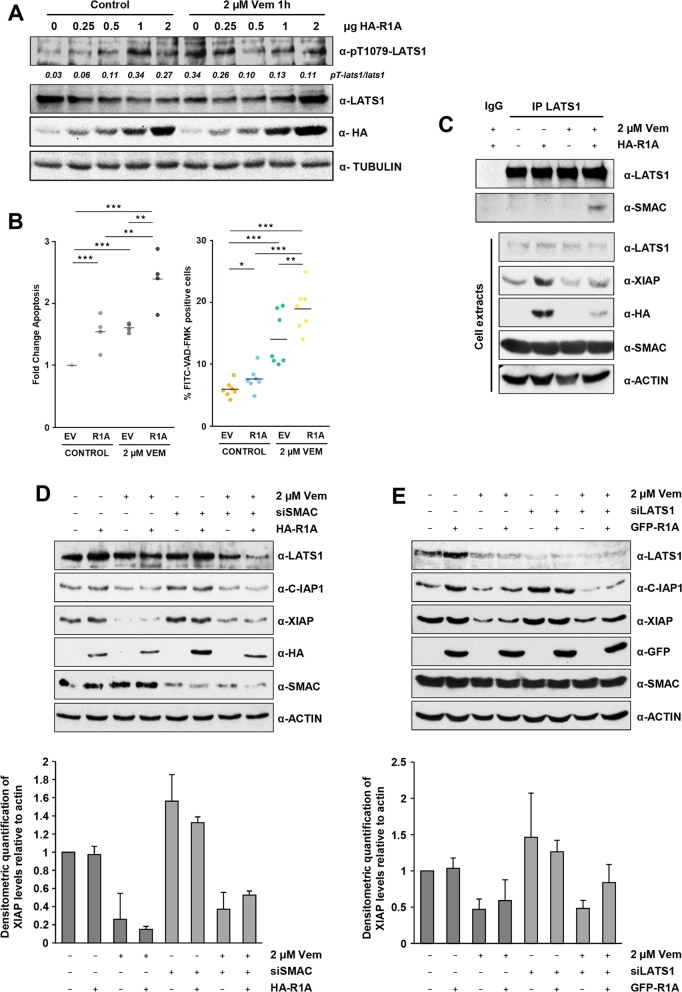
Vemurafenib enhances RASSF1A-mediated LATS1-SMAC interaction in A375 melanoma cells. **A** LATS1 phosphorylation of A375 melanoma cells upon increasing amounts of RASSF1A expression in growing conditions or after 2 µM of Vemurafenib treatment for 1 hour. Levels of transfected HA-RASSF1A and endogenous LATS1 and phospho-LATS1 (pT1079-LATS1) are shown. Values correspond to densitometric quantification of pT1079 signal vs total LATS1 levels. **B** Left: Apoptosis of A375 melanoma cells transfected with RASSF1A and treated with 2 µM of Vemurafenib for 18 hours. Percentage of apoptotic cells was assayed by flow cytometry after YoPro-PI staining. Data shows fold change of apoptosis from 4 independent experiments and bars represent averages. Right: Percentage of A375 melanoma cells showing Caspase 3 activation upon RASSF1A (R1A) overexpression and treated with 2 µM of Vemurafenib for 18 hours determined by flow cytometry after FITC-VAD-FMK staining. Statistical analysis: *t* test *p* ≤ 0.1*; *p* ≤ 0.05**; *p* ≤ 0.01***. **C** LATS1-SMAC interaction in A375 melanoma cells transfected with HA-RASSF1A upon 2 µM of Vemurafenib treatment for 10 hours assayed by co-immunoprecipitation. Levels of immunoprecipitated LATS1 and SMAC are shown (upper panel). Normal IgG was used as negative control. Total levels of LATS1, XIAP, SMAC and transfected HA-RASSF1A are shown (lower panel). Actin levels were used as loading control. **D** Effect of SMAC knockdown over IAP protein levels upon RASSF1A expression and Vemurafenib treatment. A375 melanoma cells were co-transfected with HA-RASSF1A and siRNA for SMAC (or the corresponding empty vector/non-target siRNA) as indicated and treated with 2 µM of Vemurafenib for 18 hours. Total levels of LATS1, C-IAP1, XIAP, transfected HA-RASSF1A and downregulation of SMAC levels are shown. Actin levels were used as loading control (upper panel). Fold change of XIAP total levels normalised vs actin levels are shown after densitometric quantification of 2 independent experiments ±SD (lower panel). **E** Effect of LATS1 knockdown over IAP protein levels upon RASSF1A expression and Vemurafenib treatment. A375 melanoma cells were co-transfected with GFP-RASSF1A and siRNA for SMAC (or the corresponding empty vector/non-target siRNA) as indicated and treated with 2 µM of Vemurafenib for 18 hours. Total levels of SMAC, C-IAP1, XIAP, transfected GFP-RASSF1A and downregulation of LATS1 levels are shown. Actin levels were used as loading control (upper panel). Fold change of XIAP total levels normalised vs actin levels are shown after densitometric quantification of 3 independent experiments ±SD (lower panel).

Since it was previously reported that MST1/2 kinases can be bound and inhibited by mutant BRAF [18, 19], we tested whether BRAF inhibition could trigger LATS1 proapoptotic signalling alone or in combination with RASSF1A restoration. Using LATS1 phosphorylation state as readout of LATS1 activation, we found that increasing amounts of RASSF1A slightly promoted LATS1 activity in growing conditions and that the BRAF inhibitor Vemurafenib enhanced LATS1 activation (Fig. 6A). Apoptosis assays showed that RASSF1A enhanced Vemurafenib-mediated apoptosis in A375 cells (Fig. 6B). Thus, we checked whether LATS1-mediated degradation of XIAP was regulated by BRAF inhibitors. Indeed, Vemurafenib treatment induced the LATS1-SMAC interaction upon RASSF1A expression (Fig. 6C) and a concomitant decrease in XIAP expression (Fig. 6C). To confirm that Vemurafenib-dependent changes of XIAP are dependent on LATS1 and SMAC we downregulated SMAC or LATS1 expression and checked for XIAP levels. In both cases, we found that, while Vemurafenib treatment decreases XIAP protein levels, a reduction in SMAC or LATS1 partially rescued Vemurafenib effect over XIAP (Fig. 6D, E) whereas C-IAP1 levels remained unchanged. It is worth mentioning that SMAC seems to be more important in this role compared to LATS1. These results support the importance of the LATS1-SMAC mediated degradation of XIAP upon BRAF inhibition in melanoma cells.

### Birinapant-induced C-IAP1 degradation promotes LATS1-SMAC interaction and cooperates with LATS1 to induce apoptosis

**Fig. 7:**
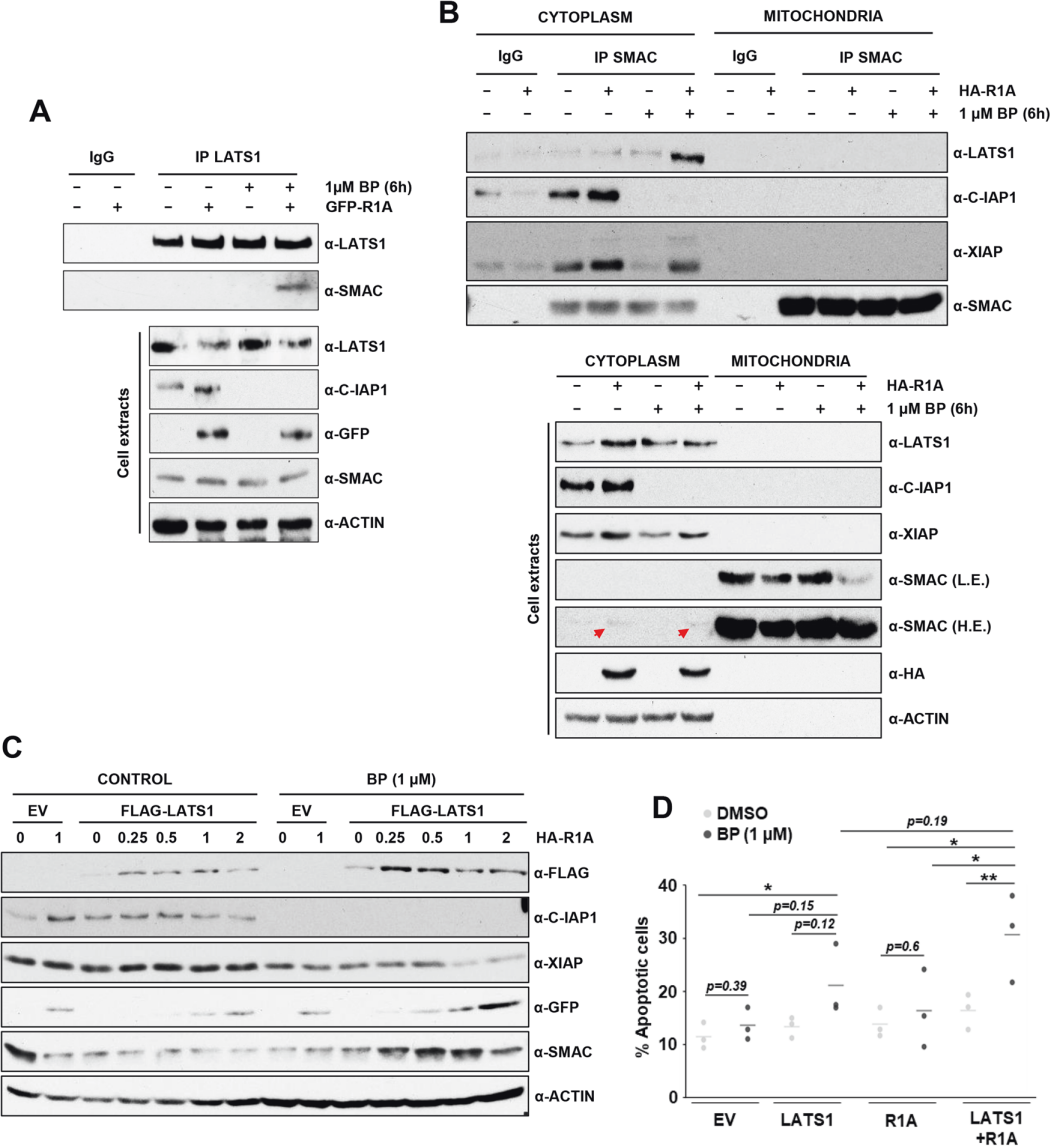
Birinapant treatment of A375 cells promotes LATS1-SMAC interaction followed by XIAP degradation and apoptosis. **A** Endogenous LATS1-SMAC interaction in A375 cells transfected with GFP-RASSF1A or the corresponding empty vector assessed by co-immunoprecipitation upon 1 µM Birinapant (BP) treatment overnight (or corresponding amount of DMSO). Endogenous LATS1 was immunoprecipitated and levels of immunoprecipitated LATS1 and SMAC are shown. Normal IgG was used as negative control for the IP (upper panel). Total levels of LATS1, C-IAP1, GFP-RASSF1A and SMAC corresponding to LATS1 immunoprecipitation are shown (lower panel). Actin levels were used as loading control. **B** Subcellular fractionation of A375 cells transfected with HA-RASSF1A or the corresponding empty vector and treated with 1 µM BP for 5 hours (or the corresponding amount of DMSO) followed by SMAC immunoprecipitation from each of the fractions. Levels of SMAC and co-immunoprecipitated LATS1, C-IAP1 and XIAP are shown (upper panel). Normal IgG was used as negative control. Total levels of LATS1, C-IAP1, XIAP, SMAC and HA-RASSF1A are shown (lower panel). Actin levels were used as loading control. **C** Total lysates corresponding to A375 cells transfected with FLAG-LATS1 and increasing amounts of GFP-RASSF1A (or corresponding empty vectors), followed by overnight starvation and 1 µM BP treatment as indicated. Levels of exogenous FLAG-LATS1 and GFP-RASSF1A and endogenous C-IAP1, XIAP and SMAC are shown. Actin levels were used as loading control. **D** Apoptosis assay of A375 cells transfected with FLAG-LATS1 and HA-RASSF1A as indicated and treated with 1 µM BP overnight. Apoptosis was measured by YoPro-PI staining. Data represent ±SD of 3 independent experiments. T.TEST: *p* ≤ 0.1*; *p* ≤ 0.5**.

Our data so far shows that IAP protein levels could determine melanoma cells sensitivity to LATS1-induced apoptosis. We studied the effect of the SMAC mimetic Birinapant (herein BP), a potent C-IAP1 antagonist that blocks C-IAP1’s SMAC binding site and promotes C-IAP1 degradation thereby preventing the binding and inactivation of SMAC. First, BP treatment promoted LATS1-SMAC interaction in the presence of RASSF1A and a complete degradation of C-IAP1 (Fig. 7A). Cytoplasm–Mitochondria fractionation of A375 cell extracts showed that SMAC release to the cytoplasm was not affected by BP treatment (Fig. 7B, lower panel) but it enhanced the LATS1-SMAC complex formation (Fig. 7B, upper panel) evidencing the importance of C-IAP1 downregulation to allow SMAC-LATS1 interaction. In the same conditions, BP treatment decreases XIAP-SMAC interaction upon RASSF1A expression (Fig. 7B, upper panel), in agreement with an increase in XIAP degradation rate. Next, we studied the regulation of IAP protein levels and apoptosis upon BP treatment by the RASSF1A-LATS1 axis showing that BP induced a complete degradation of C-IAP1 and reduced XIAP levels (Fig. 7C). Additionally, increasing RASSF1A levels cooperated with LATS1 in promoting XIAP degradation (Fig. 7C). Importantly, BP alone did not promote apoptosis (Fig. 7D) but overexpression of LATS1 clearly induced an increase of apoptosis levels. Moreover, co-expression of RASSF1A and LATS1 caused a significant increase of apoptosis in cells treated with BP (Fig. 7D). All these data together demonstrate that BP treatment cooperates with LATS1 signalling to induce apoptosis.

## Discussion

The data presented here show a direct link between the MST2 pathway and the key regulators of apoptosis SMAC and IAPs. SMAC and IAPs have opposite roles in apoptosis and reciprocally regulate their protein levels. Under survival conditions, SMAC resides inactive in the mitochondria and members of the IAP family prevent apoptosis by impairing caspase activation and targeting suboptimal cytoplasmic SMAC levels for degradation [31]. In apoptotic cells, the members of the BCL2 family BAX and BAK form pores in the outer mitochondrial membrane leading to the release of several proapoptotic proteins including SMAC [32, 33]. High levels of SMAC in the cytoplasm result in the inhibition of IAPs and subsequent activation of caspases [34]. This process is part of the apoptotic pathways and despite intense research we still lack a complete picture of the mechanisms that regulate this molecular machinery. The work described here demonstrates that LATS1 interacts with cytoplasmic SMAC following its release from the mitochondria caused by a proapoptotic signal (Fig. S3). The LATS1-SMAC complex requires the presence of XIAP, and LATS1 regulates the functions of these proteins in a kinase-independent fashion. The presence of LATS1 in this complex seems to counteract the inhibitory effect of XIAP on SMAC resulting in an increase of SMAC levels due to protein stabilisation. In turn, SMAC bound to LATS1 promotes the ubiquitination and subsequent degradation of XIAP, removing the inhibitory effect of this protein over caspases and ultimately leading to an increase in apoptosis. LATS1-induced degradation of XIAP seems to be dependent on SMAC levels. On the other hand, C-IAP1 prevents the interaction between SMAC and LATS1 in the absence of proapoptotic signals which in turn results in an accumulation of XIAP. Hence, the data shows that both IAPs contribute to the prevention of LATS1-mediated apoptosis by different mechanisms. The mechanism of how C-IAP1 modulates the LATS1-SMAC interaction is uncertain and needs further research. One simple explanation would be that releasing SMAC from C-IAP1 sequestration would make it more accessible for LATS1 binding. Importantly, our work shows that LATS1 is a signalling hub connecting the MST2 and the apoptotic pathways. The formation of the LATS1-SMAC complex is positively regulated by the tumour suppressor RASSF1A and MST2, the other core proteins of the non-canonical proapoptotic MST2 pathway. RASSF1A promotes the interaction of LATS1 and SMAC and mediates the stabilisation of this protein in the cytoplasm. Importantly, our proteomics screening has allowed us to map the effect that the MST2 pathway activation has on other proteins of the intrinsic apoptosis pathway. Moreover, the interactome study of LATS1 and SMAC shows a cluster of proteins closely related to IAPs that include TRAF1/2, TAB3 and HAX1 further supporting the idea that LATS1 is an important regulator of the apoptotic machinery. These novel LATS1 interactors might be involved in the regulation of XIAP by LATS1. In particular HAX1 is an antiapoptotic protein that promotes XIAP stability by impairing its polyubiquitination [35]. Importantly, LATS1 interacts and regulates Omi/hTra2 [36], another proapoptotic protein released from the mitochondria that have been shown to mediate the cleavage of HAX1 leading to XIAP ubiquitination and degradation [37]. Thus, one possibility would be that LATS1 promotes XIAP ubiquitination through Omi/hTra2-mediated HAX1 proteolysis.

Importantly, we also show that this new crosstalk between the MST2 pathway and SMAC occurs in different cellular systems including breast, cervical carcinoma and skin cancer cell lines. In particular, we show evidence of the relevance of this crosstalk in BRAF^V600E^ melanoma cells. The MST2 pathway role in melanoma is poorly understood and most studies have solely focussed on the role of YAP [38]. In a parallel study, we have seen that loss of expression of MST2 and LATS1 is common in melanoma cell lines that have acquired resistance to BRAF inhibitors [19]. We have demonstrated that oncogenic BRAF can inhibit MST1/2 in these cell lines and prevent the activation of its proapoptotic signal. We have also shown that LATS1 is degraded by induction of ubiquitination in melanoma cells. Here, we confirm the relationship between BRAF^V600E^, the most common driving mutation of malignant melanoma, and the MST2 pathway and show that it prevents MST2-LATS1 interaction and LATS1-dependent apoptosis. Our data indicate that the inhibitory effect of BRAF^V600E^ over the MST2 proapoptotic pathway is exacerbated by the loss of expression of RASSF1A, which is commonly reported in this cancer type [39]. We show that the loss of RASSF1A expression likely prevents SMAC from inhibiting IAPs, as the LATS1-SMAC complex is not effectively induced in these cells. Importantly, RASSF1A loss can cause the upregulation of the YAP1 target C-IAP2 further showing the relevance of the MST2 pathway in the regulation of apoptosis through IAP-SMAC [40]. These data, together with our previous findings, draw a picture where oncogenic BRAF-dependent transformation requires the silencing of the MST2 proapoptotic network, which includes the intrinsic apoptosis pathway, in addition to the activation of the well-characterised proliferative pathways such as the ERK and AKT branches [1].

Finally, we also explored the possible relevance of the novel molecular mechanisms described here for the treatment of mutant BRAF tumours. Specially interesting was the relevance to the effectiveness of novel SMAC mimetics that are currently being tested in clinical trials [41, 42]. Overexpression of IAPs have been shown in metastatic melanoma and is considered one of the factors that prevents the engagement of the apoptotic pathways in these tumours [4, 5]. These mimetics have been shown to cause limited proapoptotic effect as single agents in melanoma cell lines despite inducing C-IAP degradation [7, 9, 10]. Our data show that the SMAC mimetic Birinapant, which was designed to target C-IAP1, promotes the interaction of LATS1 and SMAC and the degradation of XIAP in A375 cells in a LATS1-dependent manner. While this increase in apoptosis does not require the expression of RASSF1A in BRAF^V600E^ mutant cells, re-expression of this tumour suppressor increases the level of apoptosis induced by LATS1. Thus, it seems that the effect of this drug is closely related to the functional activation of the MST2 pathway, and it might be important to test the patients for the expression of the core proteins of the pathway to predict the effectiveness of this treatment. Additionally, these observations also open the possibility that the combination of this drug with demethylating agents that restore RASSF1A expression have a stronger effect than single treatment with Birinapant [40].

One weakness of our study is the lack of experimental work in animal models. These experiments are warranted and will show the physiological relevance of our findings. It will be important to ascertain the role of the LATS1-SMAC crosstalk in the resistance to different treatments and how to restore this proapoptotic network with new drug combinations. Despite this limitation, we present here a detailed mechanistic characterisation of a novel regulating mechanism of the apoptotic pathways.

## Materials and methods

### Cell lines and inhibitors

All cell lines were purchased from ATCC and have been authenticated and exponentially grown at 37 °C and 5% CO_2_. HeLa and HEK293 were cultured in DMEM (Gibco, MA USA) supplemented with 10% FBS (Gibco) and 2 mM l-Glutamine (Gibco). A375, SK-Mel2, SK-Mel28, SK-Mel239 were cultured in RPMI (Gibco) supplemented with 10% FBS (Gibco) and 2 mM l-Glutamine (Gibco). Vemurafenib (PLX4032) and Birinapant were purchased from Selleck Chemicals (TX, USA).

### Transfections

Lipofectamine^TM^ 2000 (Thermo Fisher Scientific, MA, USA) was used for transfections following the manufacturer’s protocol. Briefly, cells were seeded at 60% confluency prior to transfection. The DNA (µg):Lipofectamine (µl) ratio used was 0.5:6.25 and the mix was prepared in serum-free media and incubated for 20 min at room temperature. Complete media was replaced by half volume of serum-free media and lipofectamine-DNA complexes were added dropwise. After 4–6 hours lipofectamine-containing media was replaced by complete media and kept for at least 24 hours prior to corresponding treatment. In the case of siRNA transfections, 50 pmol:6.25 µl ratio was used.

### Plasmids and siRNAs

Plasmids (all human origin): pCDNA3.1-EV pEGFP-EV (Invitrogen, USA); pCDNA-LATS1^WT^-FLAG [43]; pCDNA-LATS1^KD^-FLAG generated by direct mutagenesis as explained below; pSmac-GFP was a gift from Douglas Green (Addgene plasmid # 40881 [44]); pEGFP-RASSF1A (generous gift from Farida Latif); pCDNA-RASSF1A-FLAG, pCDNA-RASSF1A-HA [25]; pCMV-HA-Ub (HA-Ubiquitin was a gift from Edward Yeh (Addgene plasmid # 18712 [45]). siRNA from Horizon: SMAC (ON-TARGETplus Human DIABLO (56616) siRNA – SMARTpool); MST2 (ON-TARGETplus Human STK3 (6788) siRNA – SMARTpool); XIAP (ON-TARGETplus Human XIAP (331) siRNA – SMARTpool); C-IAP1 (ON-TARGETplus Human BIRC2 (329) siRNA – SMARTpool); siRNA negative control (ON-TARGETplus Non-targeting siRNA #1).

### LATS1 kinase dead generation by site-directed mutagenesis

A LATS1 kinase dead mutant (LATS1^KD^) of human origin was generated by a single point mutation at D846A using the QuikChange II Site-Directed Mutagenesis PCR kit (Agilent Technology, CA USA) according to manufacturer’s protocol. The pCDNA-LATS1^WT^-FLAG construct was used as template. Primers: Fw 5’-CATATTAAATTGACTGCCTTTGGCCTCTCTGCACTGGC-3’; Rv 5’-GCCAGTGCAAGAGGCCAAAGGCAGTCAATTTAATATG-3’. Sequence was validated by DNA sequencing.

### Western blot and immunoprecipitation

Cells were lysed as described before and analysed by western blot or immunoprecipitated as described in the supplementary documents [26, 27]. Immunoprecipitates were also used for proteomics screening where indicated. Full-length uncropped original blots are included in supplemental materials (data source file).

Cells were lysed in 1% NP40 lysis buffer: 20 mM HEPES (Sigma-Aldrich, MO, USA), 150 mM NaCl (Sigma-Aldrich), 10 mM NaF (Sigma-Aldrich), 1% NP40 (Calbiochem, CA USA), supplemented with protease and phosphatase inhibitor cocktails (Roche, Switzerland). Protein extracts were clarified by centrifugation at 14000 rpm for 15 min at 4 °C. NuPAGE LDS Sample Buffer (InvitroGene, MA USA) was added to the samples and boiled at 95 °C for 5 min. Proteins were resolved by SDS-PAGE acrylamide gels and transferred to PVDF membrane using a semi-dry transfer system (BioRad, CA, USA). Prior to incubation with primary antibodies membranes were blocked with 4% BSA for 1 hour at room temperature. Primary antibodies used (1: 1000 dilution unless indicated): LATS1 (sc-12494), BRAF (sc-5284), SMAC (sc-393118), HA (sc-805), ACTIN (sc-1616), TUBULIN (sc-8035), XIAP (sc-11426) Santa Cruz Biotechnology (CA, USA); MST2 (Ab52641), SMAC (ab32023) from Abcam (UK); LATS1 (3477 S), pT1079-LATS1 (8654 S), C-IAP1 (7065 T), GFP (2956 S), CASPASE 3 (9665) from Cell Signaling Technology (MA, USA), FLAG-HRP-conjugated (A8592, Sigma-Aldrich, MO, USA), PAN-CIAP (MAB3400 R&D Systems, MN, USA). Secondary antibodies used (1:10,000 dilution unless indicated): anti-Rabbit and anti-Mouse HRP-conjugated from Cell Signaling Technology, anti-Goat HRP-conjugated (sc-2354) from Santa Cruz Biotechnology.

### Immunoprecipitation

Protein extracts were prepared as in the previous section. For endogenous immunoprecipitation, 0.5 mg of total protein was incubated with the desired antibody or corresponding normal IgG as negative control overnight at 4 °C with rotation: LATS1 (sc-12494), BRAF (sc-5284) from Santa Cruz Biotechnology, SMAC (ab32023), Goat Isotype Control (ab37373) from Abcam, Mouse Isotype Control (61656 S), Rabbit Isotype Control (3900 S) from Cell Signaling Technology. The following morning, 5 µl of Protein G-Dynabeads (Thermo Fisher Scientific) were added to each sample and incubated for another 2 hours at 4 °C with rotation. Immunocomplexes bound to the Dynabeads were washed three times using ice-cold 0.5% NP40 washing buffer (20 mM HEPES, 150 mM NaCl, 0.5% NP40) by placing the samples in a magnet and discarding supernatant after each wash. Proteins were eluted from the Dynabeads in 30 µl of 1× NuPAGE LDS Sample Buffer (InvitroGene) at 95 °C for 10 min and resolved by SDS-PAGE as described above. For anti-FLAG or anti-GFP exogenous immunoprecipitations, (ANTI-FLAG® M2 Affinity Gel, Sigma-Aldrich) or anti-GFP- (GFP-Trap®_MA, Chromotek, Germany) conjugated beads were added to 0.5 mg of protein extracts and incubated for 4 hours at 4 °C with rotation. Similarly, beads were washed three times with ice-cold 0.5% washing buffer by spinning down the samples at max speed for 30 sec and discarding supernatant. Lysis/washing buffer for LATS1-FLAG/SMAC-GFP exogenous interaction: 1% NP40, 20 mM HEPES, 300 mM NaCl, 1 mM EDTA, 1 mM EGTA, 10 mM NaF.

### Cytoplasm–mitochondria fractionation

The Qproteome^TM^ Mitochondria Isolation Kit (Qiagen, Germany) was used to isolate cytoplasmic and mitochondrial extracts according to manufacturer’s protocol. Briefly, cells were harvested, washed with 0.9% NaCl solution and pellet resuspended in ice-cold Lysis Buffer and incubated 10 min at 4 °C on an end-over-end shaker. Lysate was centrifuged at 1000 × *g* for 10 min at 4 °C and supernatant containing cytoplasmic proteins was collected in a clean tube. Cell pellet was resuspended in ice-cold Disruption Buffer and further disrupted using a Dounce homogeniser. Lysate was centrifuged at 1000 × *g* for 10 min at 4 °C, supernatant containing the mitochondrial fraction was transferred to a clean tube and centrifuge at 6000 × *g* for 10 min at 4 °C. The mitochondria-containing pellet was lysed in 1% NP40 lysis buffer. Protein extracts corresponding to the cytoplasmic and mitochondrial fractions were further analysed by western blot or subjected to immunoprecipitation assays as described above.

### Apoptosis assay by YoPro-PI staining

Percentage of apoptotic cells was measured by YoPro-PI staining followed by Flow Cytometry analysis as described before [23]. Both floating death cells and attached cells were collected and washed with ice-cold PBS. ~5 × 10^5^ cells were resuspended in 0.5 mL of PBS containing 0.5 µM of YO-PRO™-3 Iodide (InvitroGene) and incubated for 15 min on ice protected from light. Propidium Iodide (PI) (Biolegend, USA) was added (0.25 µg/mL) and incubated for 5 min on ice protected from light. Samples were acquired and analysed with a BD Accuri^TM^ C6 Flow Cytometer (MA, USA). Yo-Pro-3 fluorescence was exited using a 640 nm laser and detected with the FL4 675/25 nm filter. PI fluorescence was excited using a 488 nm laser and detected with the FL2 585/40 nm filter.

### SubG_1_ detection by PI staining

Percentage of cells in SubG_1_ phase was determined by DNA content measurement using PI staining followed by Flow Cytometry analysis as described before [25]. Both floating death cells and attached cells were collected and washed with ice-cold PBS. Cells were fixed in 70% ethanol added dropwise while vortexing and incubated at 4 °C for 1 h. Cells were centrifuged and washed twice with ice-cold PBS to remove ethanol and resuspended in PBS containing 0.5 µg/mL of PI (Biolegend, CA USA) and incubated at room temperature for 30 min protected from light. Samples were acquired and analysed with a BD Accuri^TM^ C6 Flow Cytometer. PI fluorescence was excited using a 488 nm laser and detected with the FL2 585/40 nm filter.

### Caspase 3 activation by flow cytometry

Percentage of cells showing Caspase 3 activation was determined by FITC-VAD-FMK staining, followed by flow cytometry quantification as previously described [46]. Briefly, both floating death cells and attached cells were collected, centrifuged 300 × *g* and resuspended in 5 µM FITC-VAD-FMK (CaspACE™ FITC-VAD-FMK, Promega) containing serum-free media and incubated at 37 °C for 30 minutes protected from light. Cells were washed once with PBS, resuspended in PBS containing 0.5 µg/mL of PI (Biolegend, CA USA) and incubated at room temperature for 10 min protected from light. Samples were acquired and analysed with a BD Accuri^TM^ C6 Flow Cytometer (MA, USA). FITC-VAD-FMK fluorescence was exited using a 488 nm laser and detected with the FL1 525/25 nm filter. PI fluorescence was excited using a 488 nm laser and detected with the FL2 585/40 nm filter.

### Statistical analysis

Statistical analyses were performed using Excel. The number of biological replicates was presented by individual data points in each graph and centre values indicate means. Error bars show standard deviation and *p* values were determined by Student’s *T* test (two-side) and significance between samples is denoted as *p* ≤ 0.1*; *p* ≤ 0.05**; *p* ≤ 0.01***.

### Affinity purification-mass spectrometry (AP-MS)

LATS1 and SMAC interactome was determined as previously described [27] and the extended explanation can be found in the supplementary documents (Supp Fig. 2). Briefly, cells were transfected with desired tagged constructs (FLAG-LATS1, GFP-SMAC, HA-RASSF1A) and serum-deprived overnight. Cell lysis and immunoprecipitation were performed as described above. ~1 mg of protein extract was incubated with 5 µL of ab-conjugated beads. After three washes with 0.5% NP40 washing buffer, two extra washes were performed with detergent-free washing buffer to remove detergent (20 mM HEPES, 150 mM NaCl). Immunoprecipitated complexes were trypsin-digested on-beads. First, samples were incubated in 60 µL EBI (2 M Urea, Sigma-Aldrich, 50 mM Tris-HCl, Sigma-Alrdrich pH 7.5, 5 µg/mL Trypsin Sequencing Grade, Promega, WI, USA) at 600 rpm and 26 °C for 30 min followed by centrifugation at 14000 rpm at 4 °C for 30 sec. Supernatants were collected and beads containing peptides were further eluted by adding 2 × 25 µL EBII (2 M Urea, 50 mM Tris-HCl pH 7.5, 1 mM DTT, Sigma-Aldrich), vortexed and centrifuged. Supernatants were collected and added to the previous elution rounds and left overnight at room temperature for further digestion. Then, 20 µL of 50 mM IAA (Sigma-Aldrich) were added, mixed and incubated for 30 min at room temperature. C18 stage tips were prepared as previously described (Rappsilber et al 2007) and activated by loading 50 µL of 50% AcN (Thermo Fisher Scientific)—0.1% TFA (Sigma-Aldrich). Stage tips were centrifuged at 5800 rpm for 30 sec and flow-through discarded. Next, stage tips were washed with 50 µL of 0.1% TFA and flow-through discarded. Prior to loading the digested samples to the C18 stage tips, TFA was added to each sample (final concentration 1% TFA). Samples were loaded into the C18 stage tips, flow-through discarded and washed twice with 50 µL 0.1% TFA. Peptides were eluted in clean tubes by adding twice 25 µL of 50% AcN—0.1% TFA. Finally, samples were evaporated in a CentriVap Concentrator. LCMSMS Method (Bruker timsTof Pro): Samples were run on a Bruker timsTof Pro mass spectrometer (Bruker, MA, USA) connected to a Bruker nanoElute nano-lc chromatography system. Tryptic peptides were resuspended in 0.1% formic acid. Each sample was loaded onto an Aurora UHPLC column (25 cm × 75 μm ID, C18, 1.6 μm; IonOpticks, Australia) and separated with an increasing acetonitrile gradient over 30 minutes at a flow rate of 300 nl/min. The mass spectrometer was operated in positive ion mode with a capillary voltage of 1500 V, dry gas flow of 3 l/min and a dry temperature of 180 °C. All data were acquired with the instrument operating in trapped ion mobility spectrometry (TIMS) mode. Trapped ions were selected for ms/ms using parallel accumulation serial fragmentation (PASEF). A scan range of (100–1700 m/z) was performed at a rate of 10 PASEF MS/MS frames to 1 MS scan with a cycle time of 1.15 s. Data Analysis—Maxquant [47]. The raw data were searched against the *Homo sapiens* subset of the Uniprot Swissprot database (reviewed) using the search engine Maxquant (release 1.6.11.0) using specific parameters for trapped ion mobility spectra data-dependent acquisition (TIMS DDA). Each peptide used for protein identification met specific Maxquant parameters, i.e., only peptide scores that corresponded to a false discovery rate (FDR) of 0.01 were accepted from the Maxquant database search. The normalised protein intensity of each identified protein was used for label-free quantitation (LFQ). Average of LFQ intensities per sample type were calculated and statistical analyses carried out in excel. A cutoff ≥2 was used to determine specific interactors per condition when comparing experimental sample with negative controls (T.test *p* ≤ 0.05). For differentially regulated interactors among different conditions cutoff ≤0.5 or ≥2 was used to determine those interactors upregulated or downregulated (*T* test *p* ≤ 0.05).

## Supplementary information

Editable file for table included in Figure 1A

Data source

Supplementary figures

Supplemental table 1

Supplemental table 2

## Data Availability

Initial proteomics datasets used in this project have been published before [19, 23, 27] and are available in PRIDE repository. The original data sets described in this manuscript are deposited in PRIDE repository [48], access number PXD032781.
