# Supplementary material for: Interaction of LATS1 with SMAC links the MST2/Hippo pathway with apoptosis in an IAP-dependent manner
Source: Cell Death Dis. 2022 Aug 8;13(8):692. doi: 10.1038/s41419-022-05147-3 (PMC9360443; doi:10.1038/s41419-022-05147-3)
Supplement: Supplementary file 1 — Editable file for table included in Figure 1A [file 41419_2022_5147_MOESM1_ESM.docx]

| **Protein names** | **Gene names** | **Uniprot ID** | **10% serum**  LFQ intensity | **0% Serum** LFQ intensity | **% Sequence coverage** |
| --- | --- | --- | --- | --- | --- |
| Second mitochondria-derived activator of caspase; Diablo isoform 1 variant | DIABLO SMAC | Q9NR28-1 | 0 | 5033100 | 4.1 |
