# Supplementary material for: Interaction of LATS1 with SMAC links the MST2/Hippo pathway with apoptosis in an IAP-dependent manner
Source: Cell Death Dis. 2022 Aug 8;13(8):692. doi: 10.1038/s41419-022-05147-3 (PMC9360443; doi:10.1038/s41419-022-05147-3)
Supplement: Supplementary file 2 — Data source [file 41419_2022_5147_MOESM2_ESM.pdf]

Figure 1

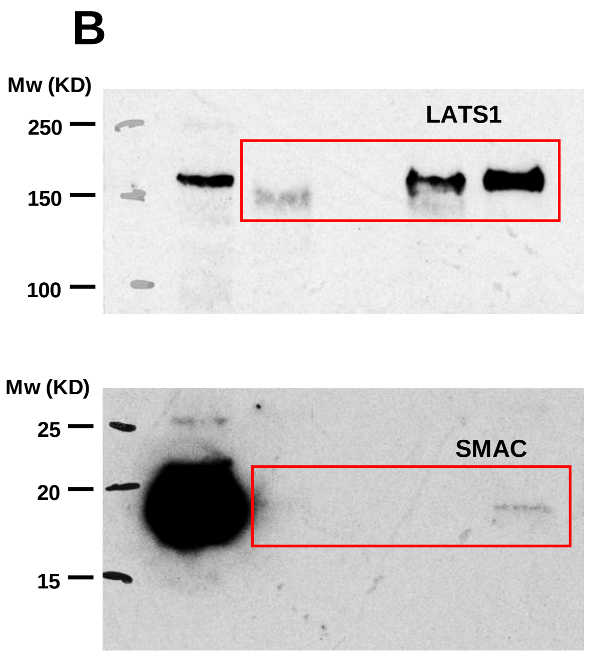
B
Mw (KD)
250
150
100
LATS1
Mw (KD)
25
20
15
SMAC

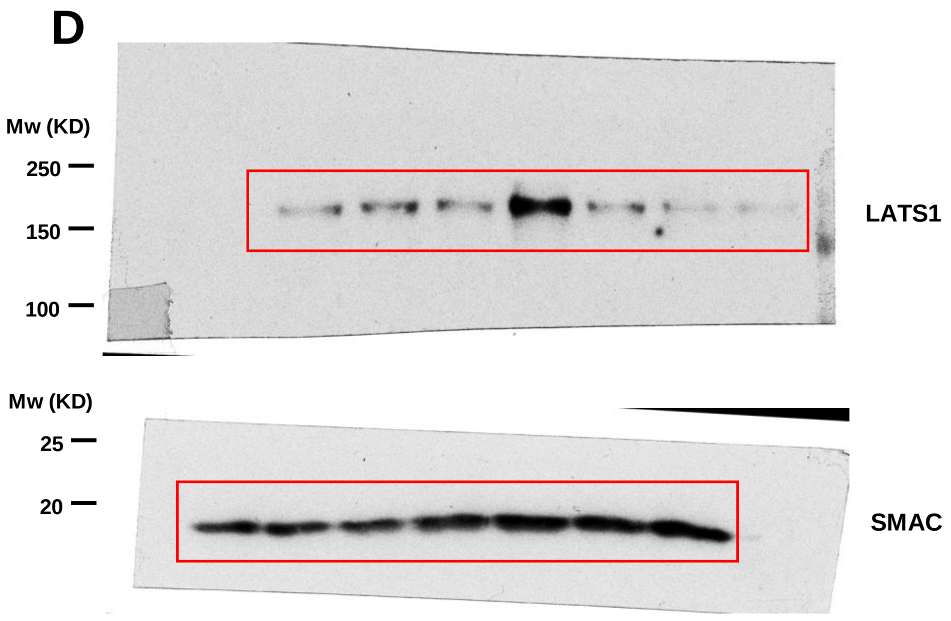
D
Mw (KD)
250
150
100
LATS1
Mw (KD)
25
20
SMAC

Figure 1

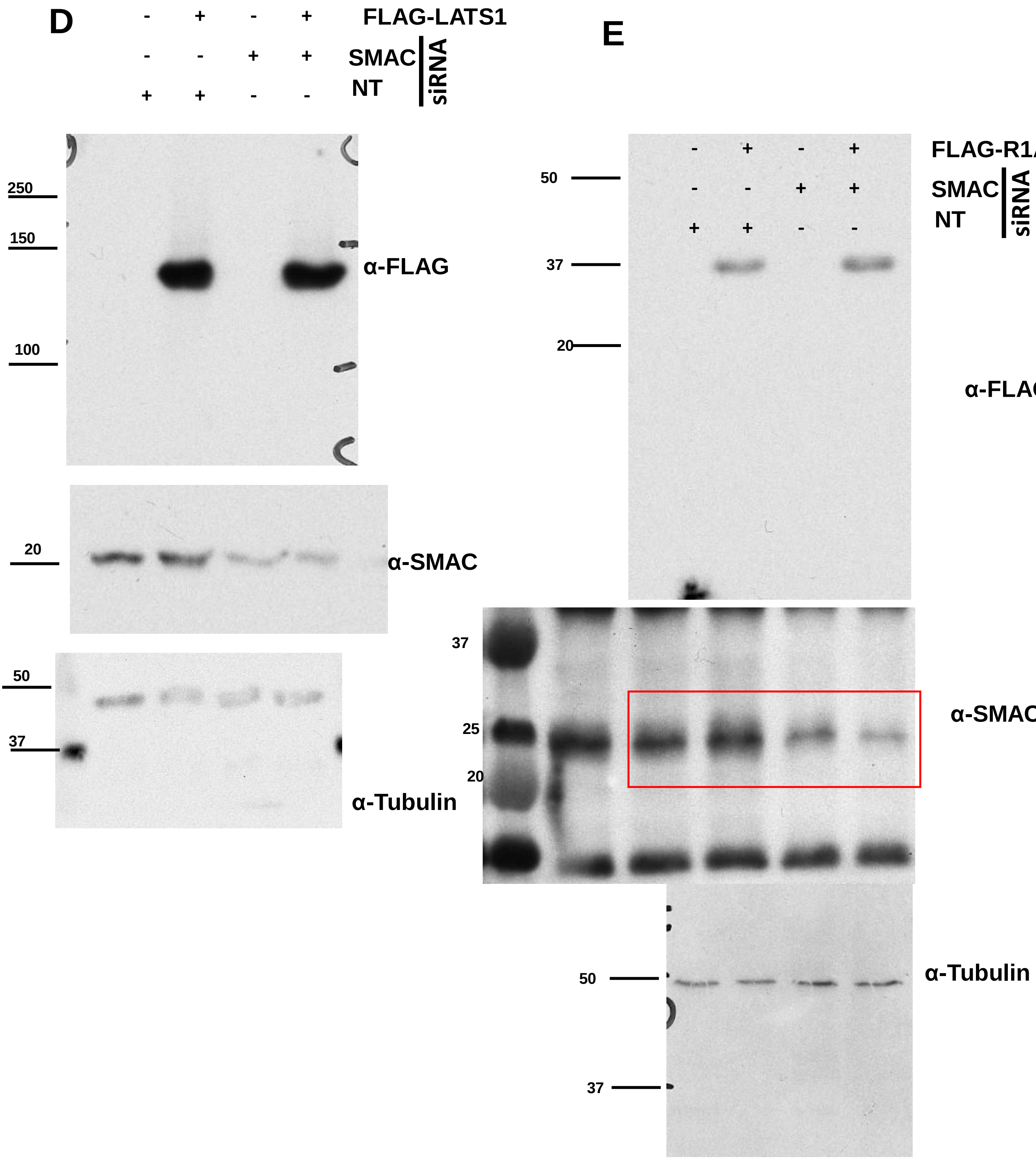
D
- + - + FLAG-LATS1
- - + + SMAC
+ + - - NT
siRNA
250
150
100
α-FLAG
20
α-SMAC
50
37
α-Tubulin
E
- + - + FLAG-R1A
- - + + SMAC
+ + - - NT
siRNA
50
37
20
α-FLAG
37
25
20
α-SMAC
50
37
α-Tubulin

Figure 2 C

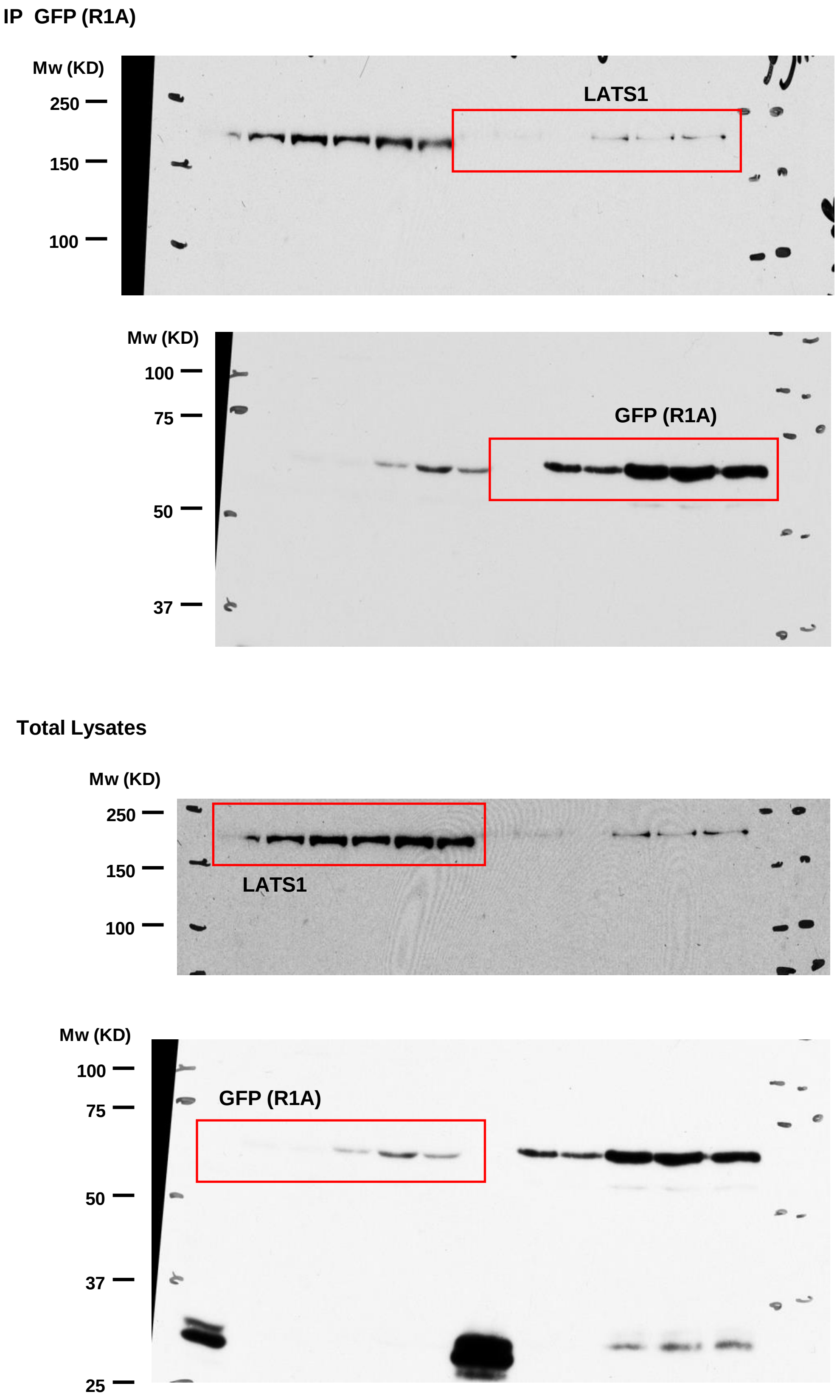
IP GFP (R1A)
Mw (KD)
250
150
100
LATS1
Mw (KD)
100
75
50
37
GFP (R1A)
Total Lysates
Mw (KD)
250
150
100
LATS1
Mw (KD)
100
75
50
37
25
GFP (R1A)

Figure 2

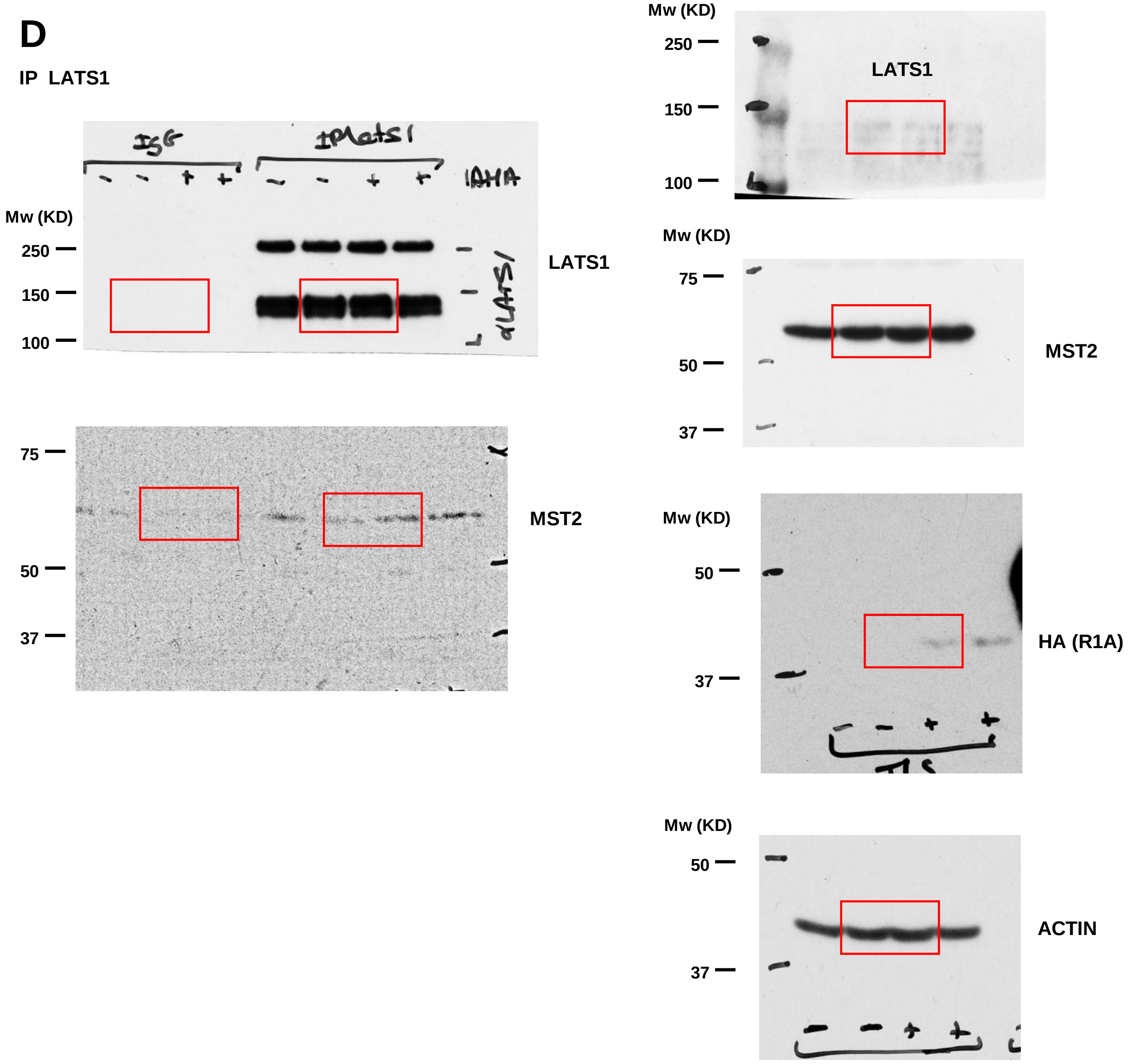
D
IP LATS1
Mw (KD)
250
150
100
LATS1
75
50
37
MST2
Total Lysates
Mw (KD)
250
150
100
LATS1
Mw (KD)
75
50
37
MST2
Mw (KD)
50
37
HA (R1A)
Mw (KD)
50
37
ACTIN

Figure 2 E

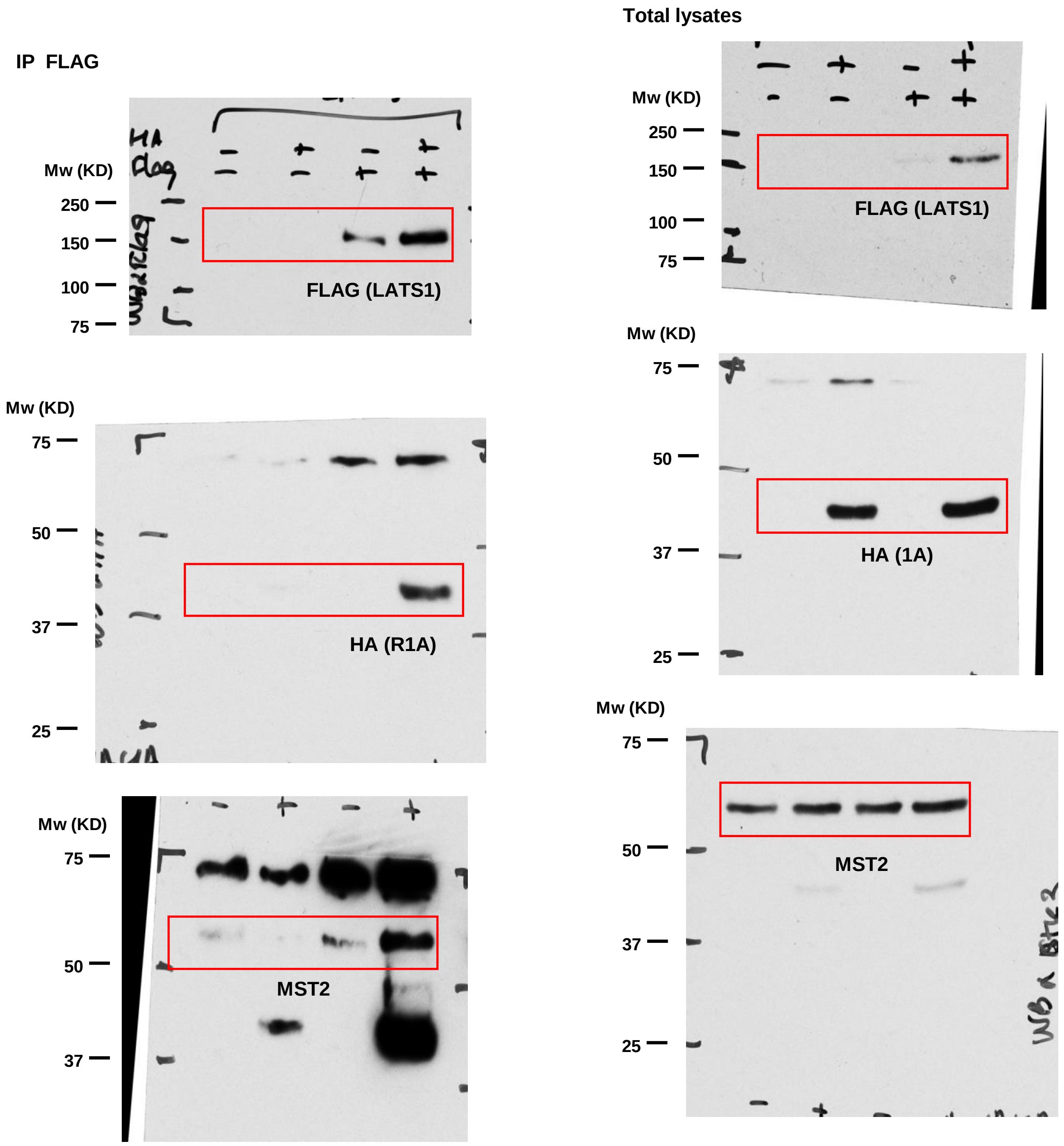
IP FLAG
Total lysates
Mw (KD)
250
150
100
75
50
37
25
HA
flag
FLAG (LATS1)
HA (R1A)
MST2
HA (1A)
FLAG (LATS1)
MST2

Figure 2

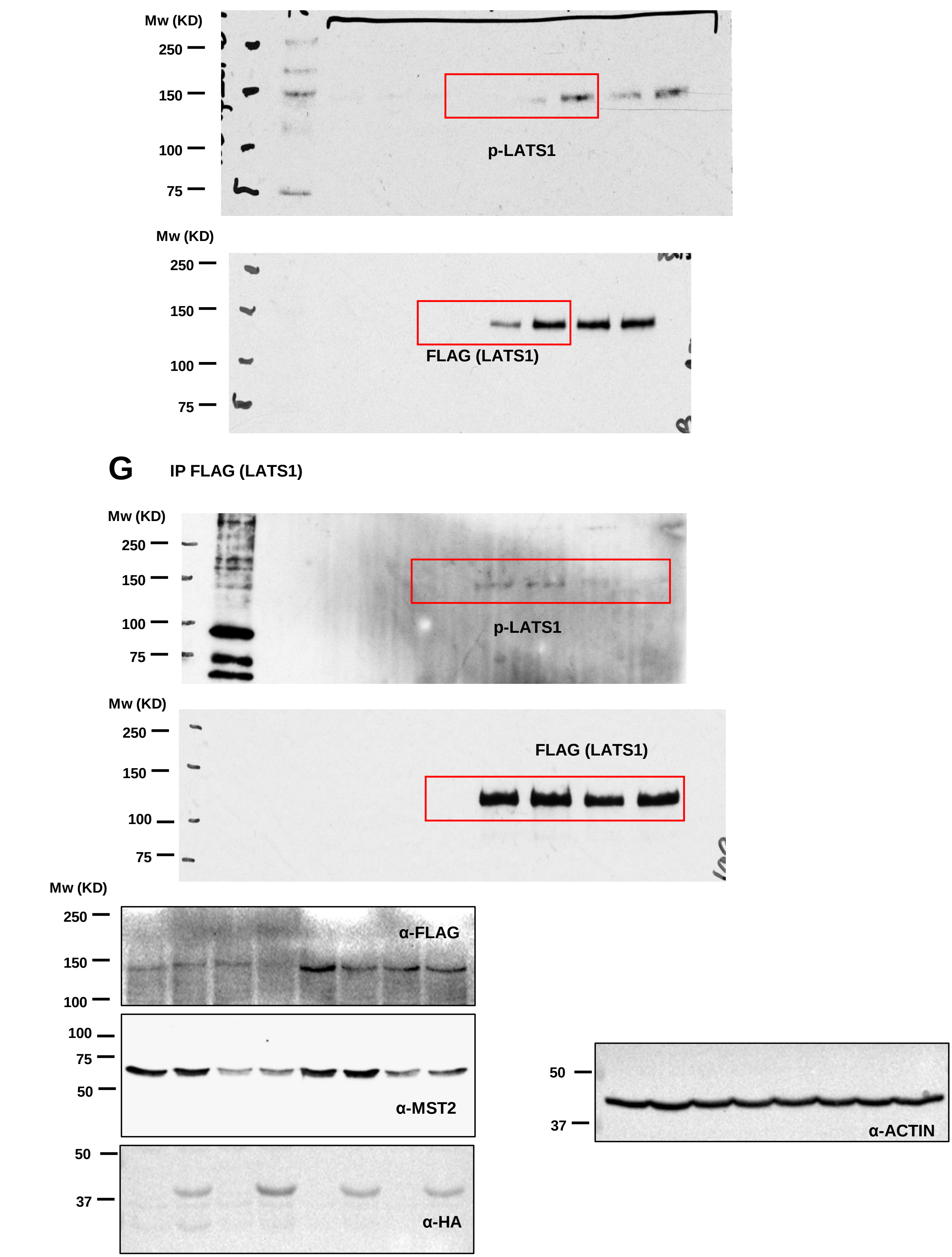
F
IP FLAG (LATS1)
Mw (KD)
250
150
100
75
p-LATS1
Mw (KD)
250
150
100
75
FLAG (LATS1)
G
IP FLAG (LATS1)
Mw (KD)
250
150
100
75
p-LATS1
Mw (KD)
250
150
100
75
FLAG (LATS1)
Mw (KD)
250
150
100
α-FLAG
100
75
50
α-MST2
50
37
α-HA
50
37
α-ACTIN

Figure 3

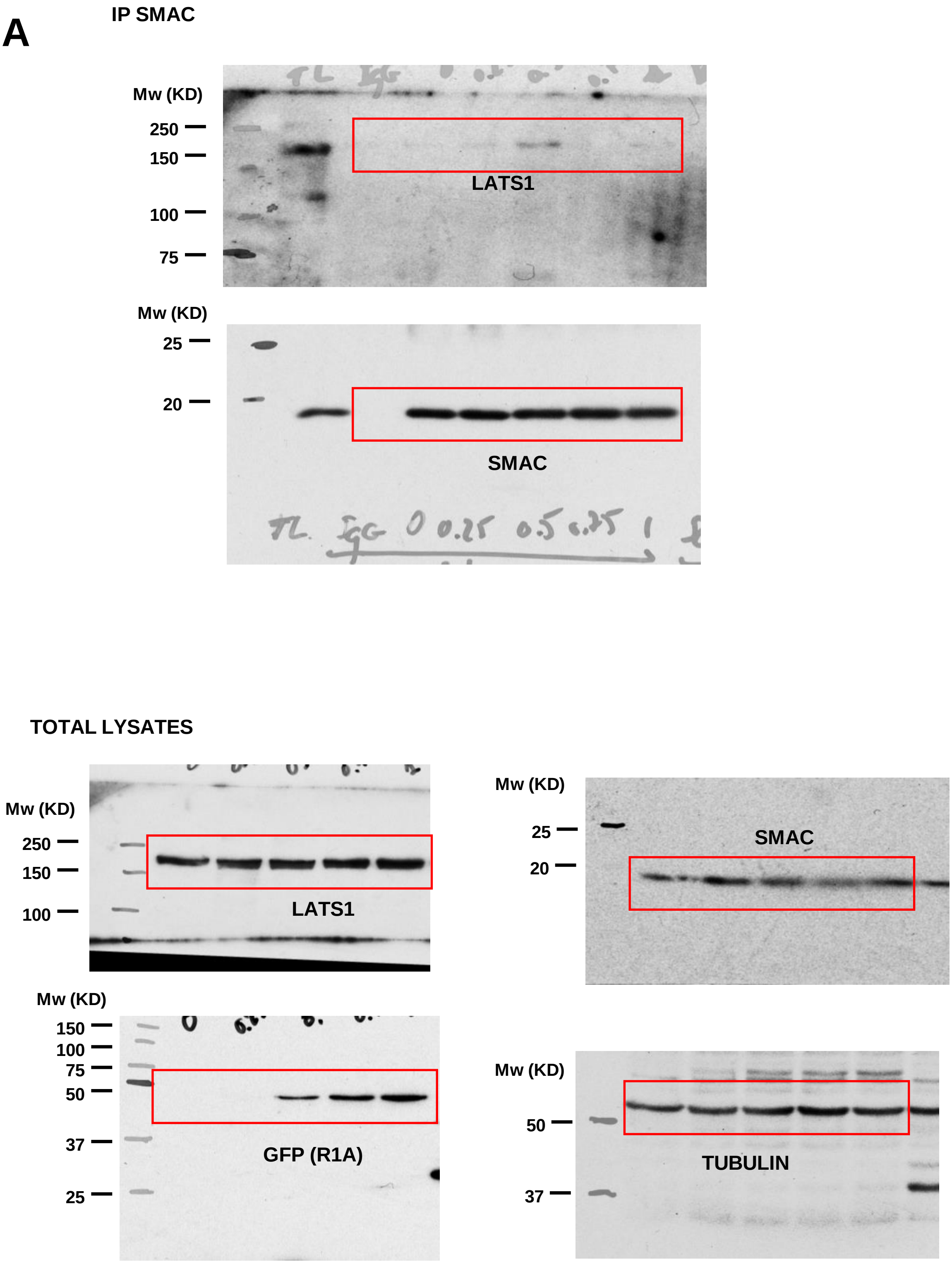

A
IP SMAC
Mw (KD)
250
150
100
75
LATS1
Mw (KD)
25
20
SMAC
TL IgG 0 0.25 0.5 0.75 1
TOTAL LYSATES
Mw (KD)
250
150
100
LATS1
Mw (KD)
25
20
SMAC
Mw (KD)
150
100
75
50
37
25
GFP (R1A)
Mw (KD)
50
37
TUBULIN

Figure 3

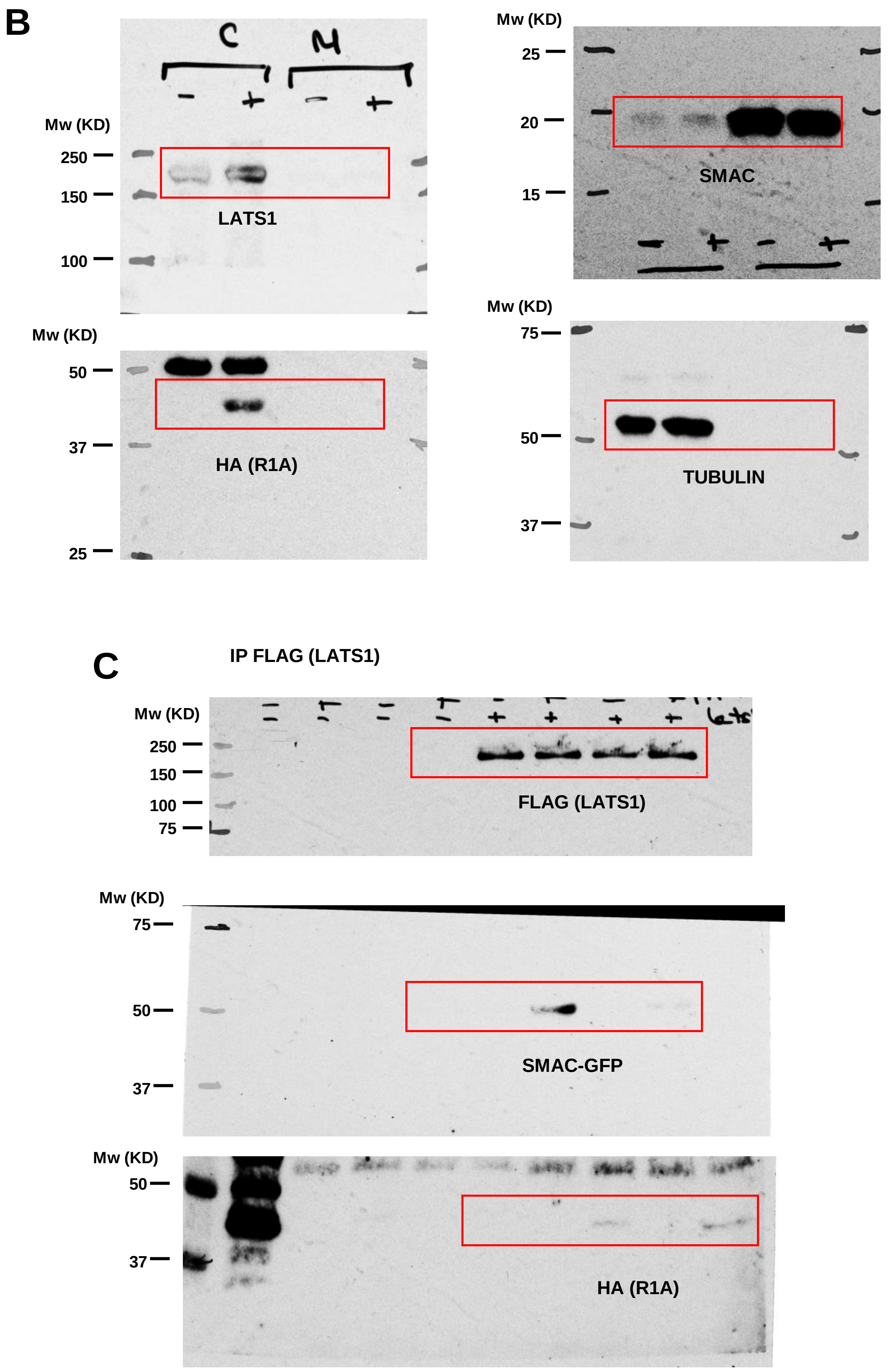
B
C
M
Mw (KD)
250
150
100
LATS1
50
37
25
HA (R1A)
20
15
SMAC
75
TUBULIN
C
IP FLAG (LATS1)
FLAG (LATS1)
SMAC-GFP
HA (R1A)

Figure 3

C

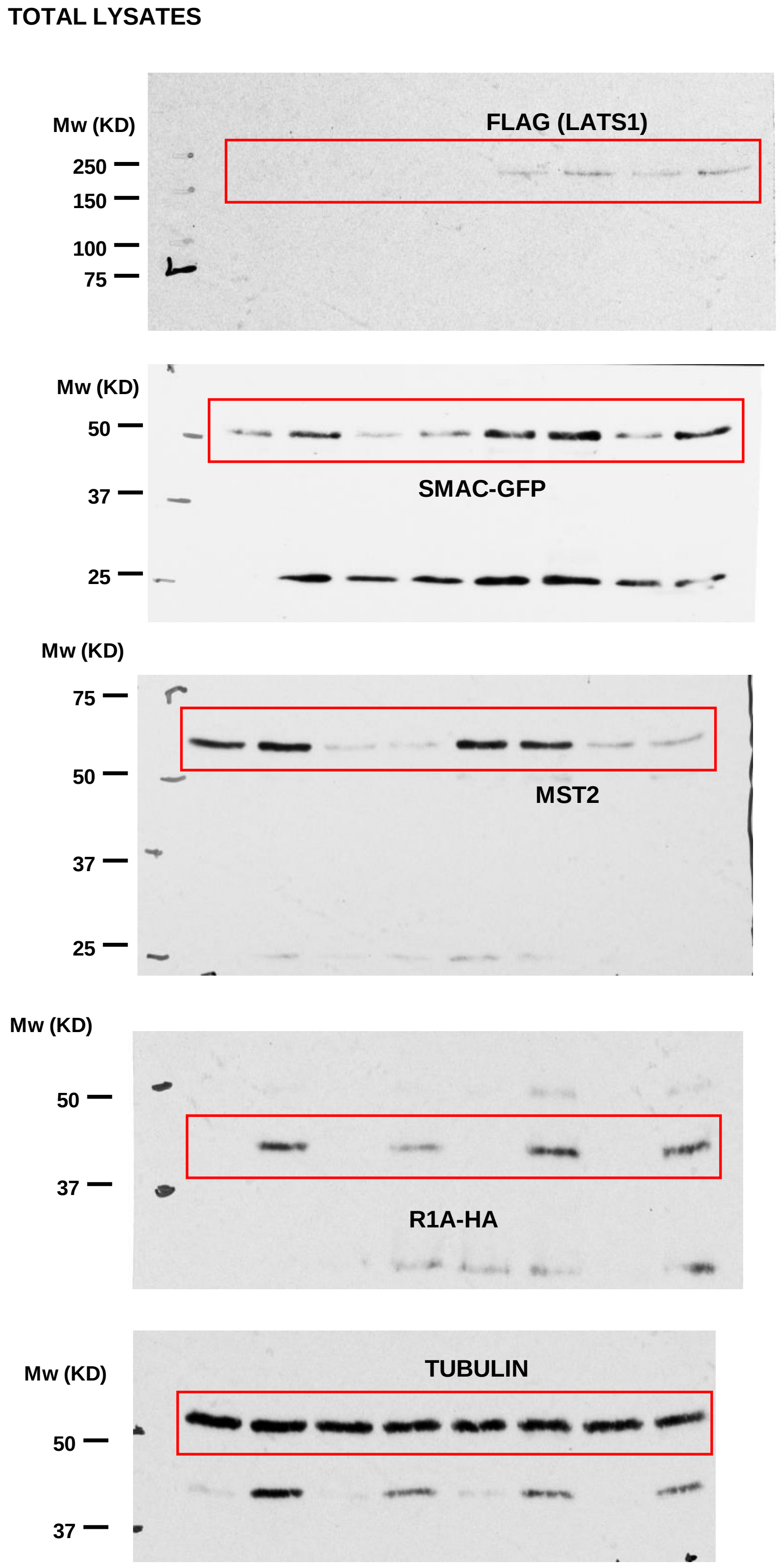
TOTAL LYSATES
Mw (KD)
FLAG (LATS1)
250
150
100
75
Mw (KD)
50
37
25
SMAC-GFP
Mw (KD)
75
50
37
25
MST2
Mw (KD)
50
37
R1A-HA
Mw (KD)
TUBULIN
50
37

D

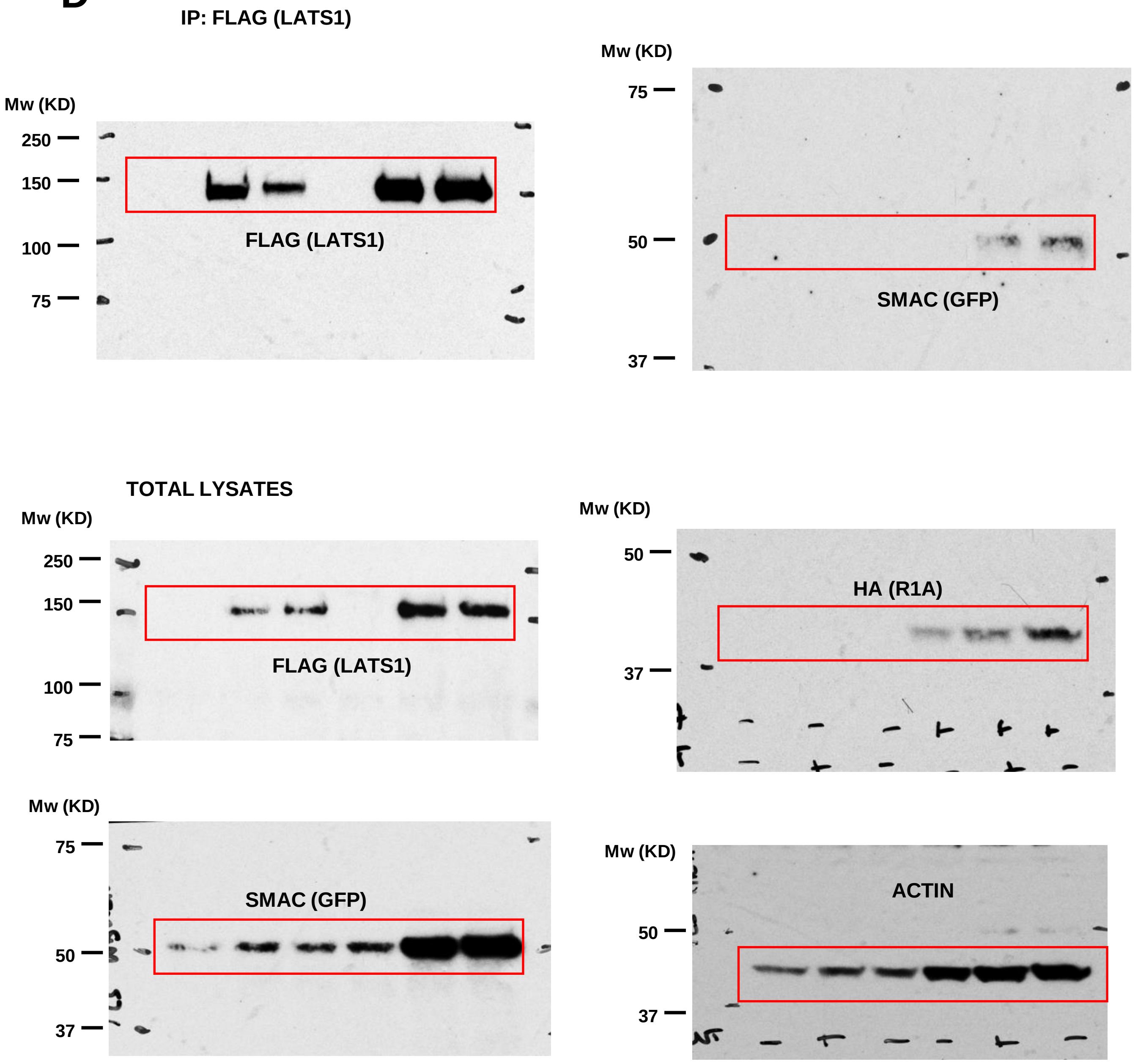
IP: FLAG (LATS1)
Mw (KD)
250
150
100
75
FLAG (LATS1)
Mw (KD)
75
50
37
SMAC (GFP)
TOTAL LYSATES
Mw (KD)
250
150
100
75
FLAG (LATS1)
Mw (KD)
50
37
HA (R1A)
Mw (KD)
75
50
37
SMAC (GFP)
Mw (KD)
50
37
ACTIN

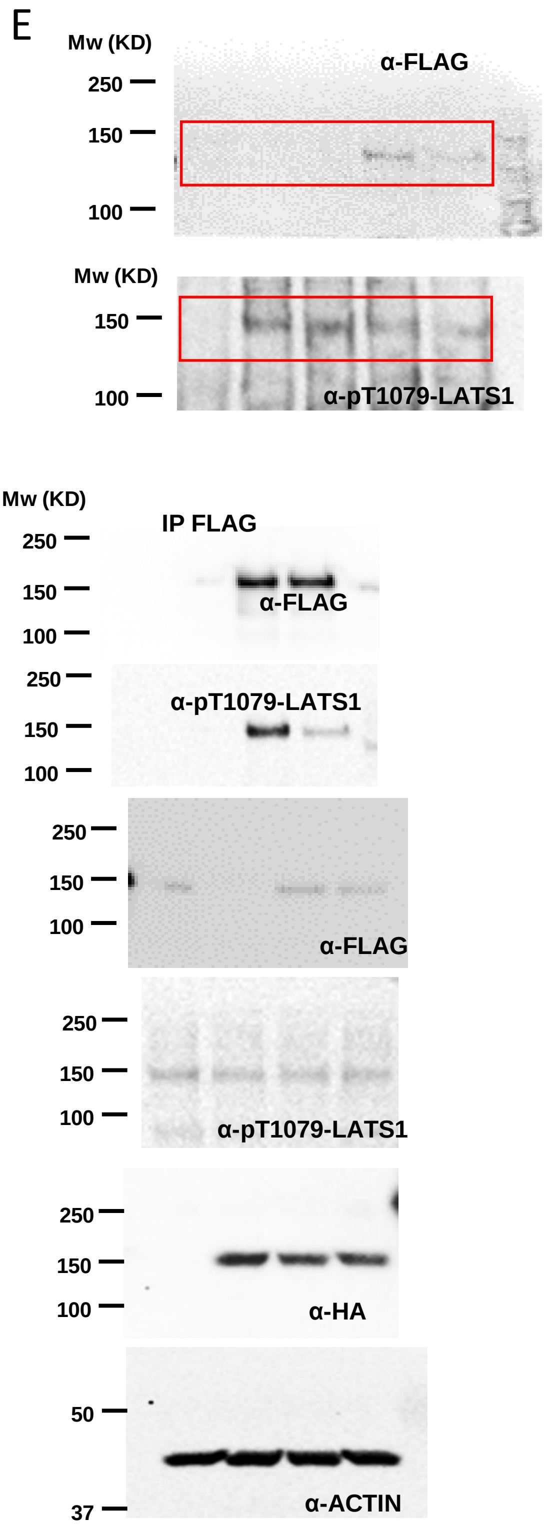
E
Mw (KD)
250
150
100
α-FLAG
Mw (KD)
150
100
α-pT1079-LATS1
Mw (KD)
IP FLAG
250
150
100
α-FLAG
250
150
100
α-pT1079-LATS1
250
150
100
α-FLAG
250
150
100
α-pT1079-LATS1
250
150
100
α-HA
50
37
α-ACTIN

Supplementary Figure 1

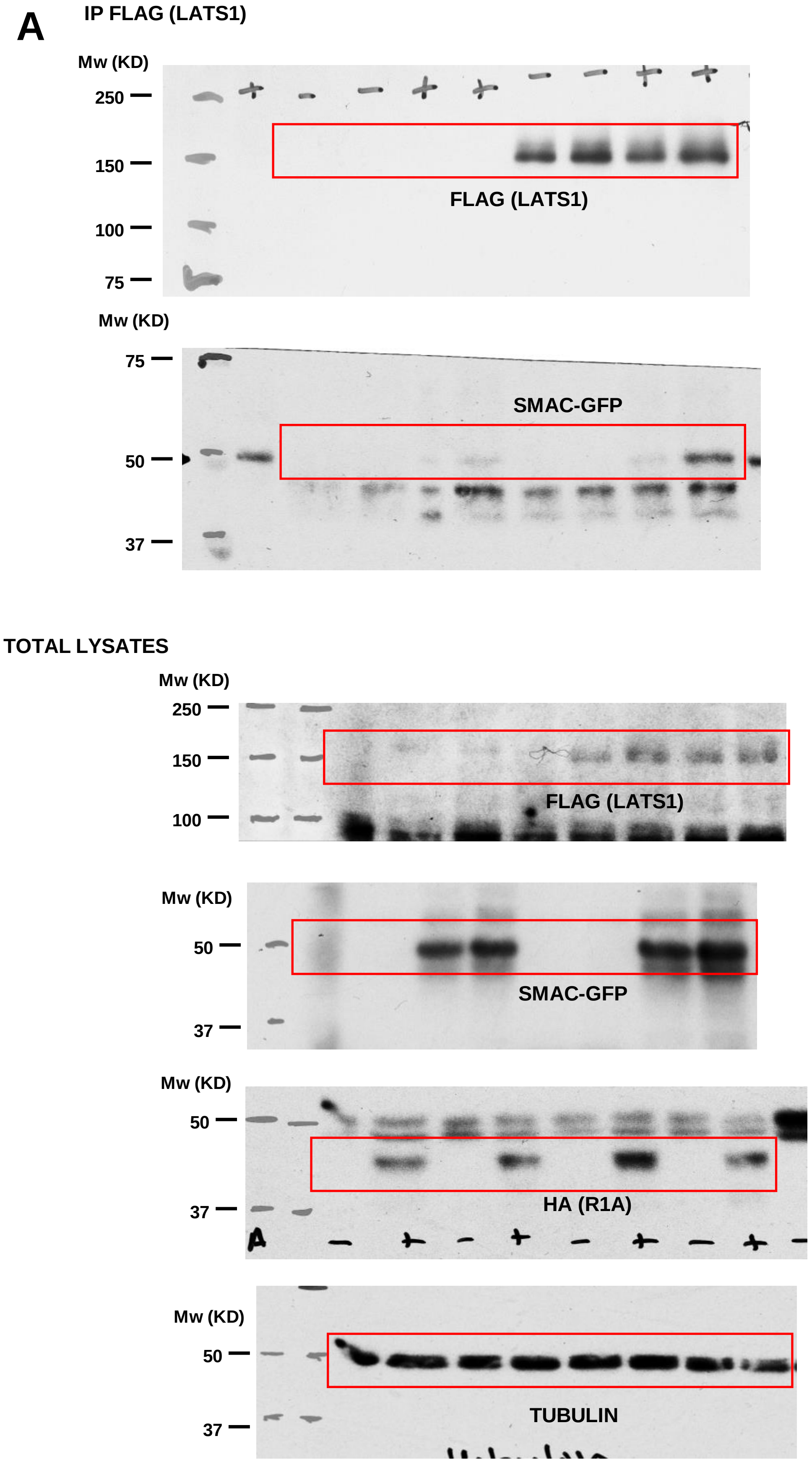

Figure 5

B

IP FLAG (LATS1)

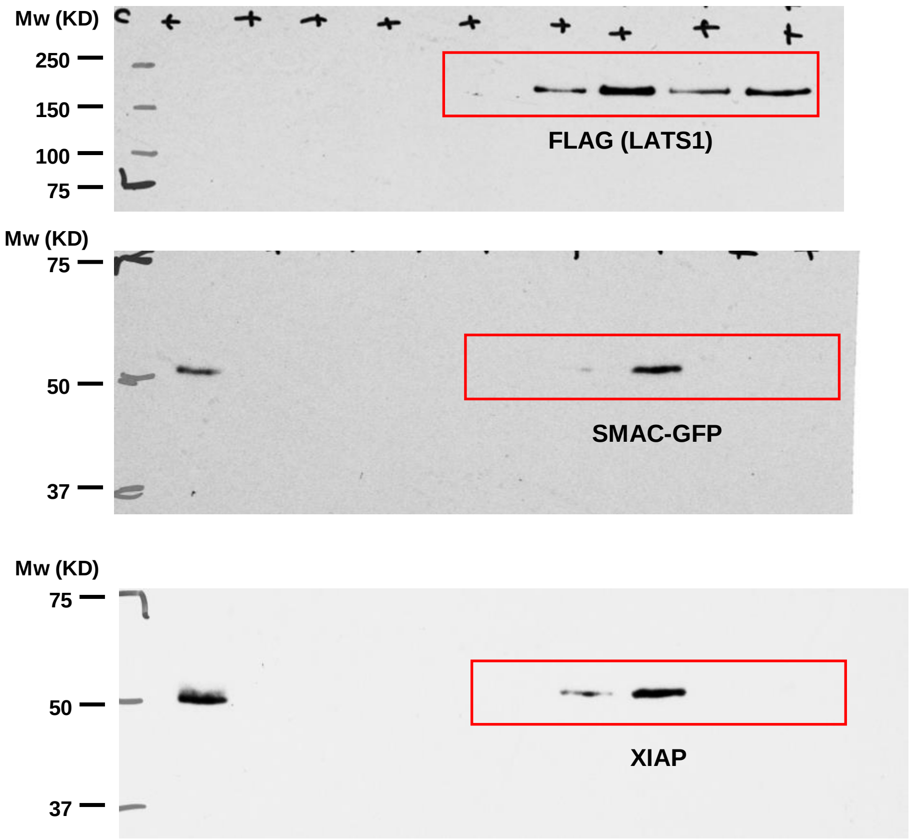

Figure 5 C

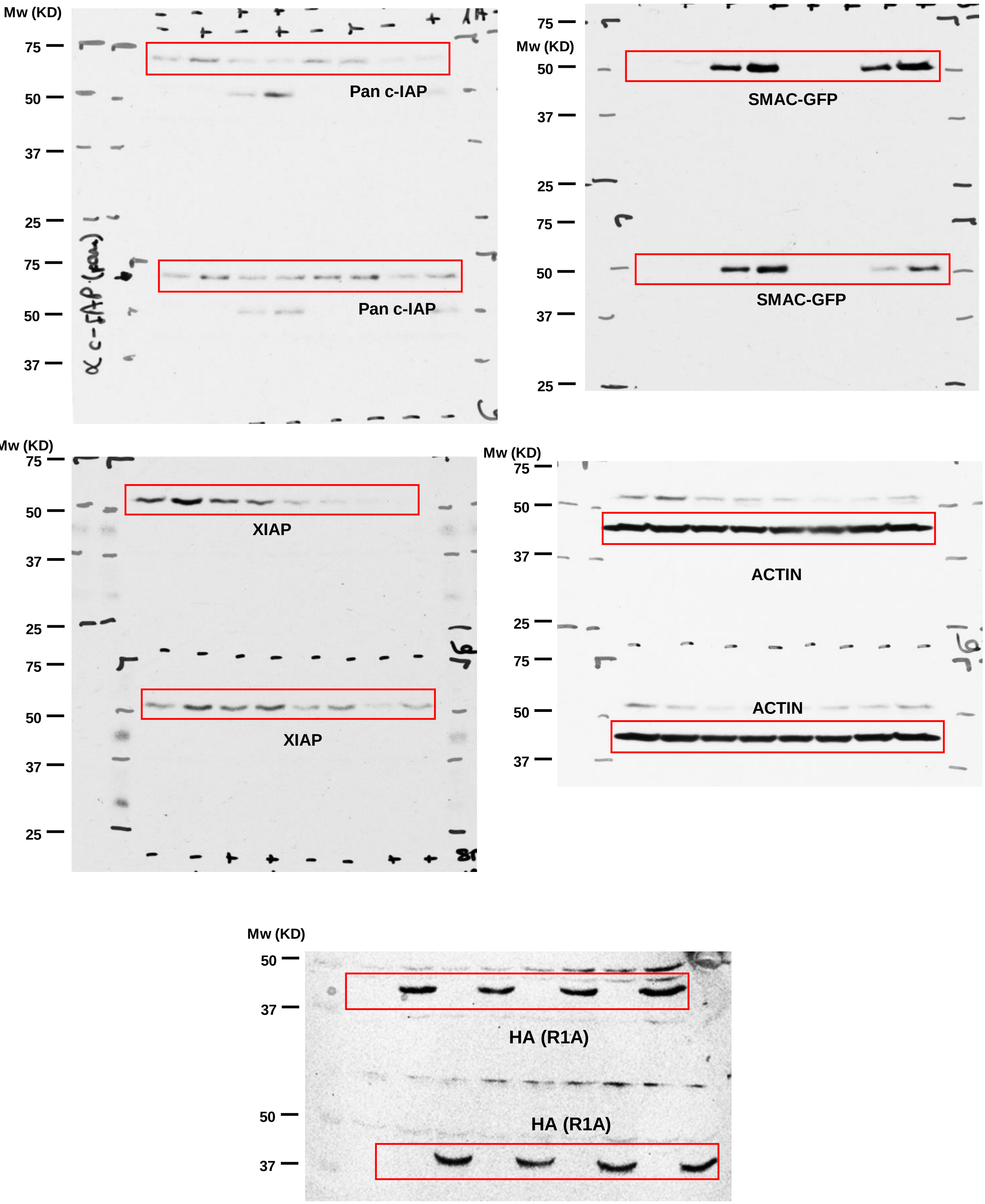
Mw (KD)
75
50
37
25
75
50
37
Pan c-IAP
Pan c-IAP
Mw (KD)
75
50
37
25
75
50
37
25
SMAC-GFP
SMAC-GFP
Mw (KD)
75
50
37
25
75
50
37
25
XIAP
XIAP
Mw (KD)
75
50
37
25
75
50
37
ACTIN
ACTIN
Mw (KD)
50
37
50
37
HA (R1A)
HA (R1A)

Figure 5

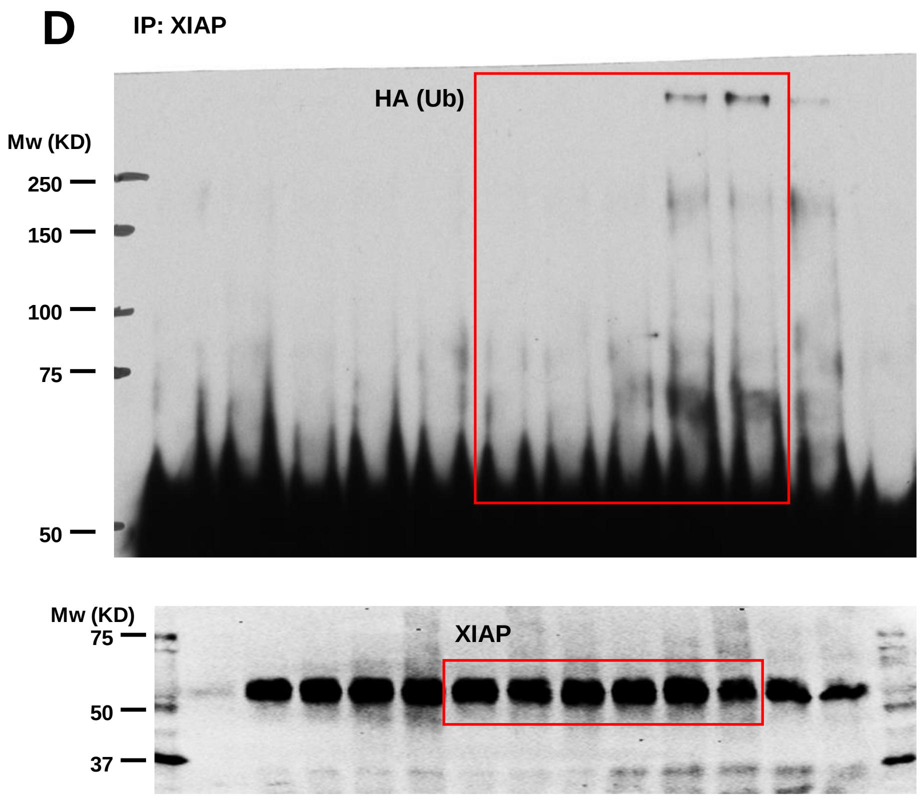
D
IP: XIAP
HA (Ub)
Mw (KD)
250
150
100
75
50
Mw (KD)
XIAP
75
50
37

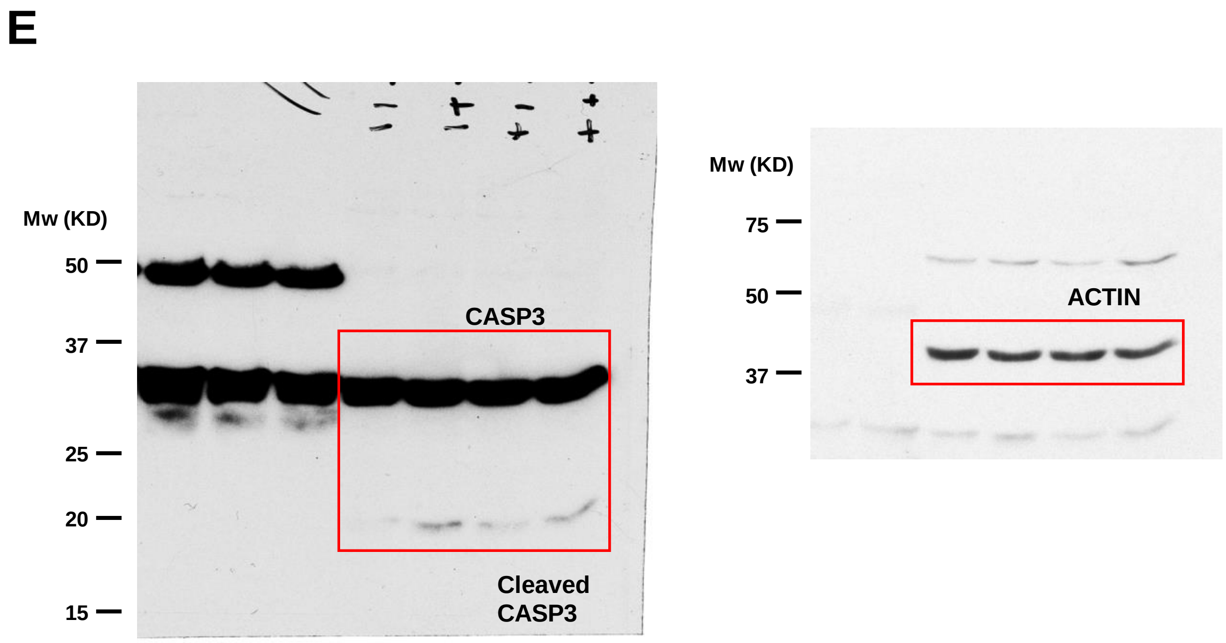
E
Mw (KD)
50
37
25
20
15
CASP3
Cleaved CASP3
Mw (KD)
75
50
37
ACTIN

Figure 5 F

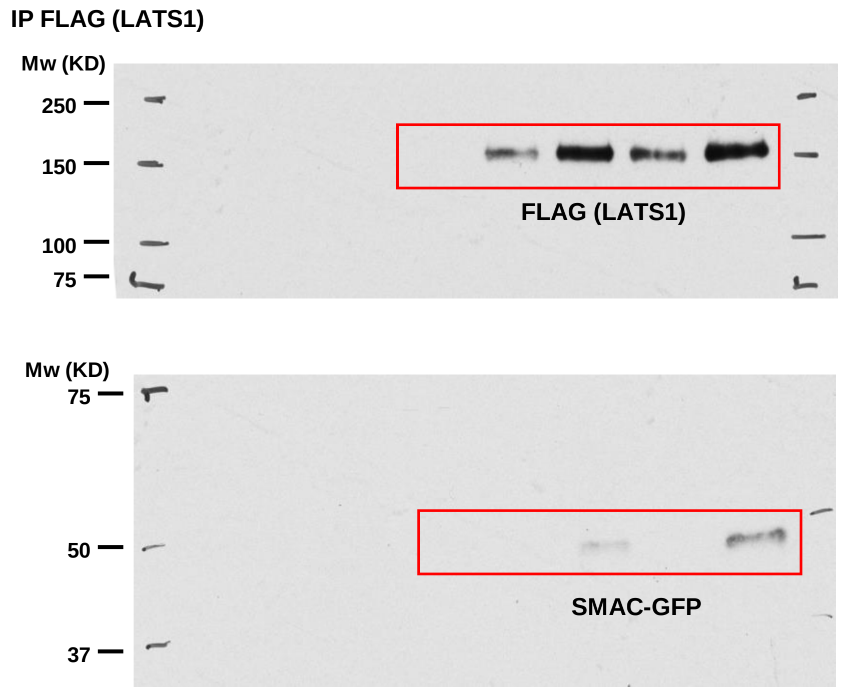
IP FLAG (LATS1)
Mw (KD)
250
150
100
75
FLAG (LATS1)
Mw (KD)
75
50
37
SMAC-GFP

G

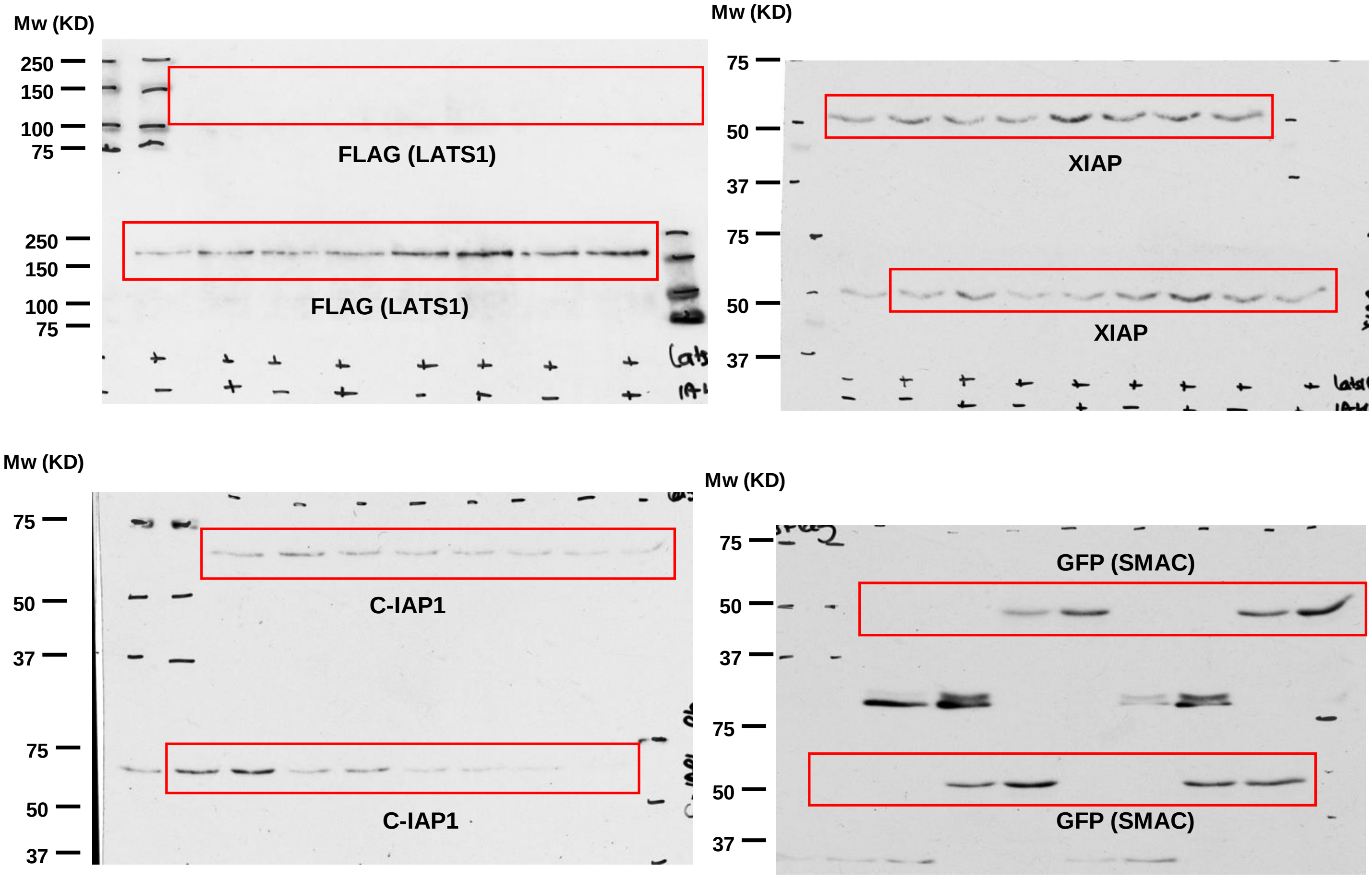
Mw (KD)
250
150
100
75
FLAG (LATS1)
250
150
100
75
FLAG (LATS1)
Mw (KD)
75
50
37
XIAP
75
50
37
XIAP
Mw (KD)
75
50
37
C-IAP1
75
50
37
C-IAP1
Mw (KD)
75
50
37
GFP (SMAC)
75
50
37
GFP (SMAC)

G *(continues from previous page)*

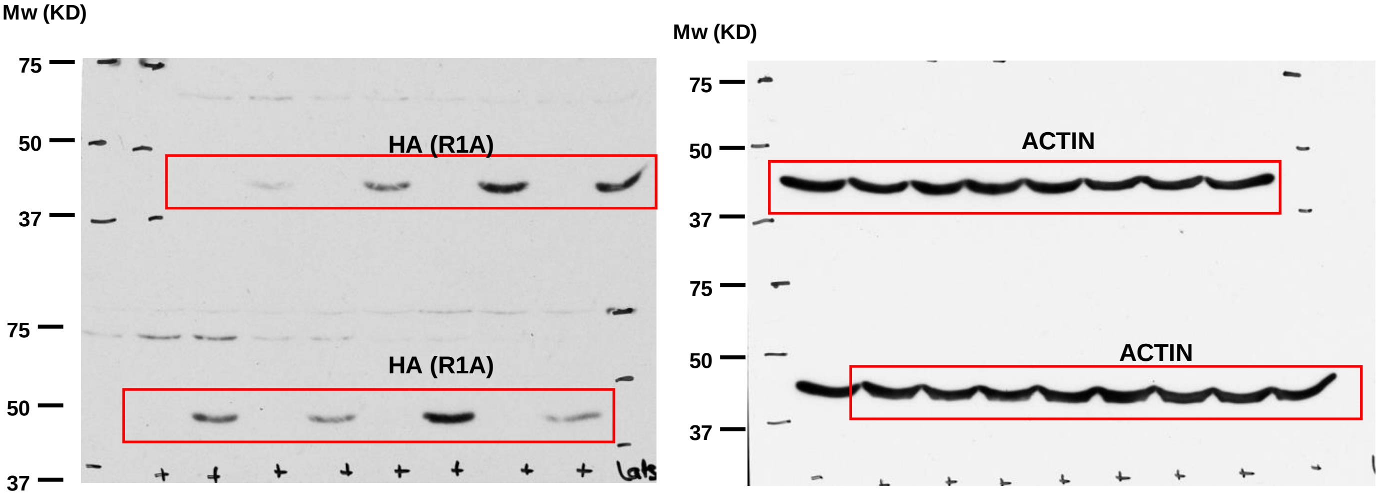

A

Figure 6

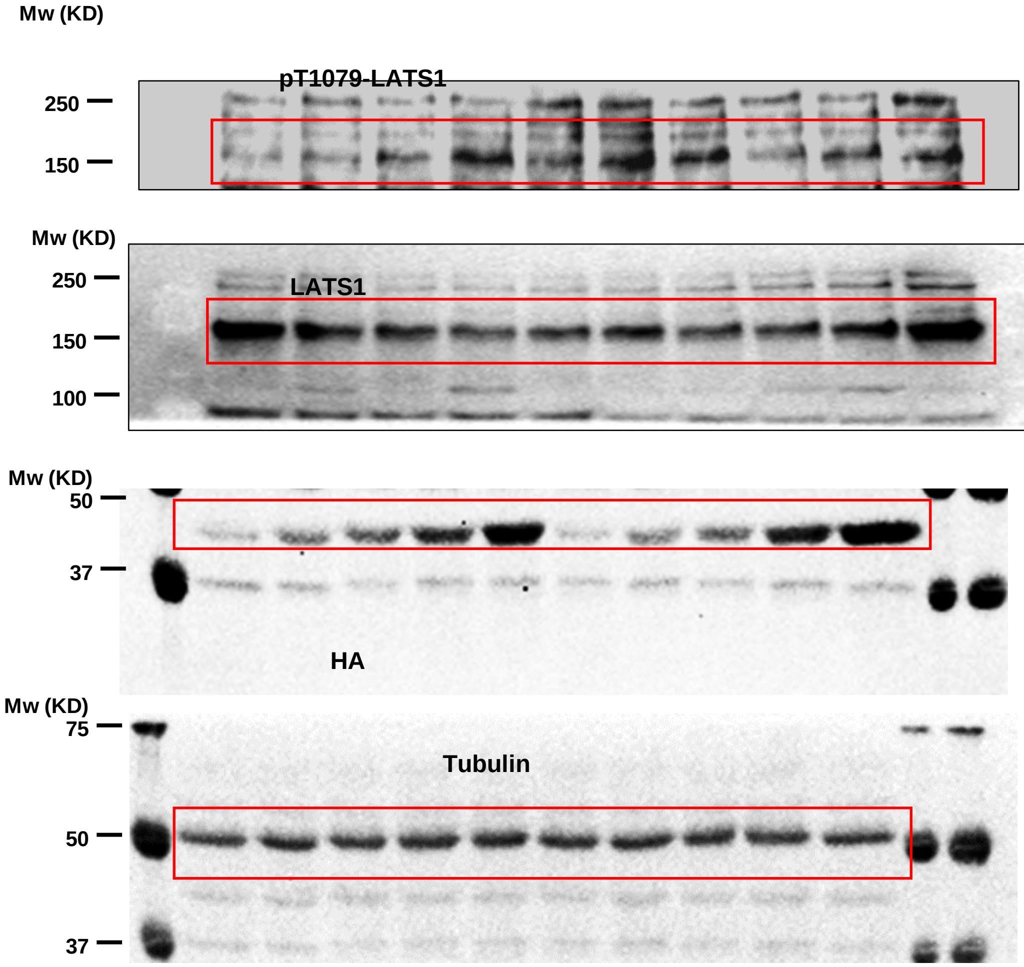

Figure 6 C

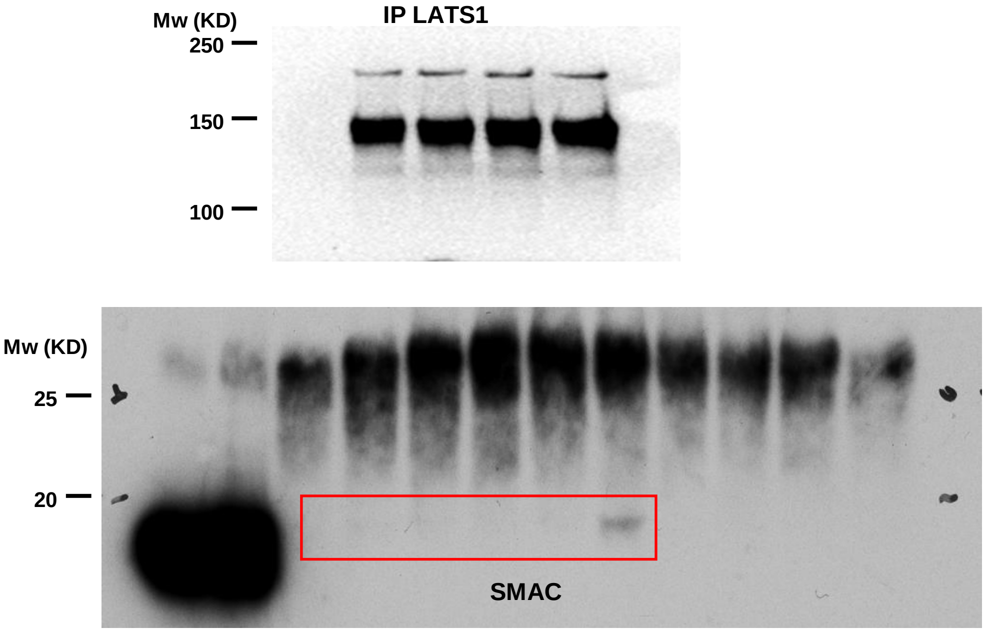

TOTAL LYSATES

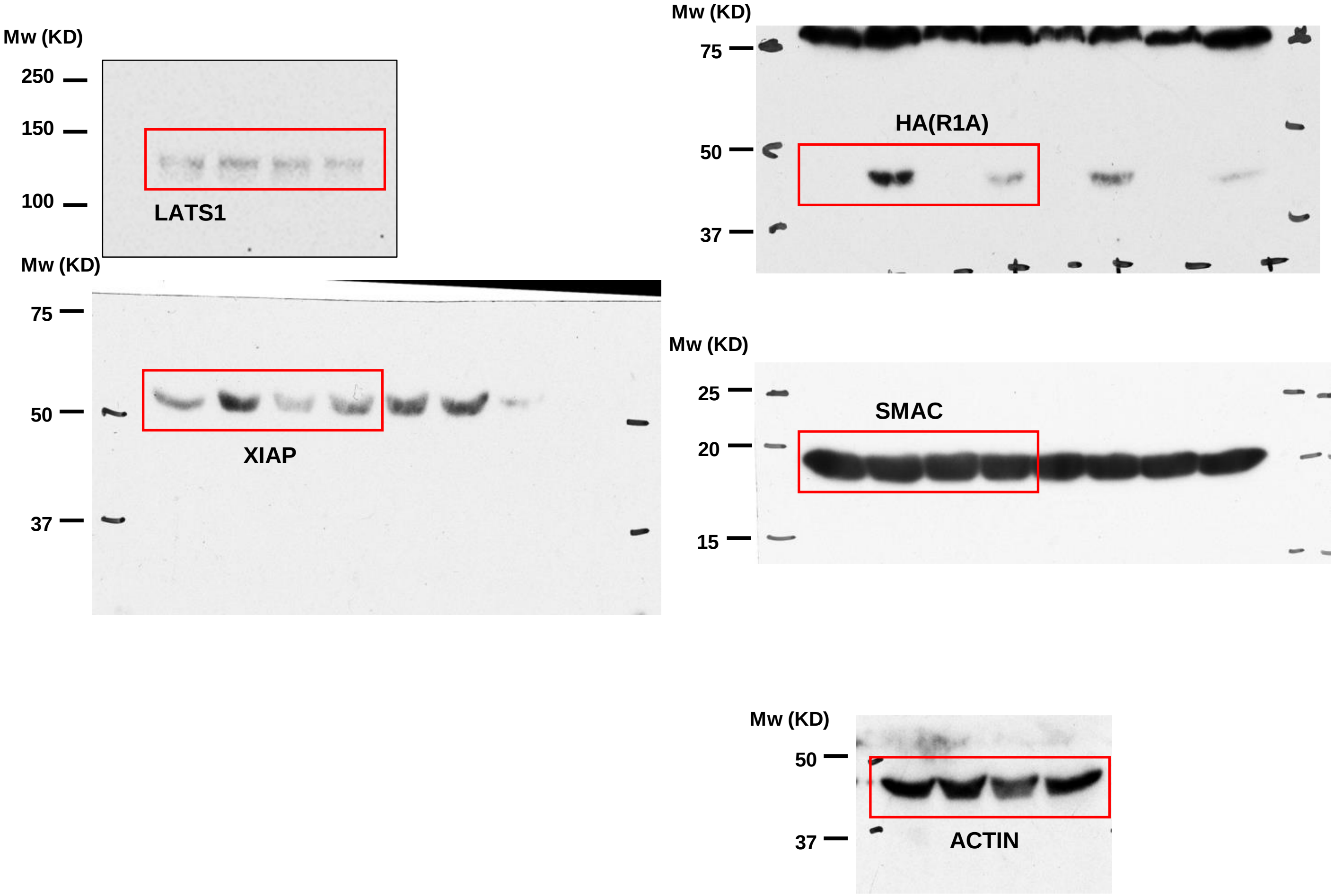

Figure 6 D

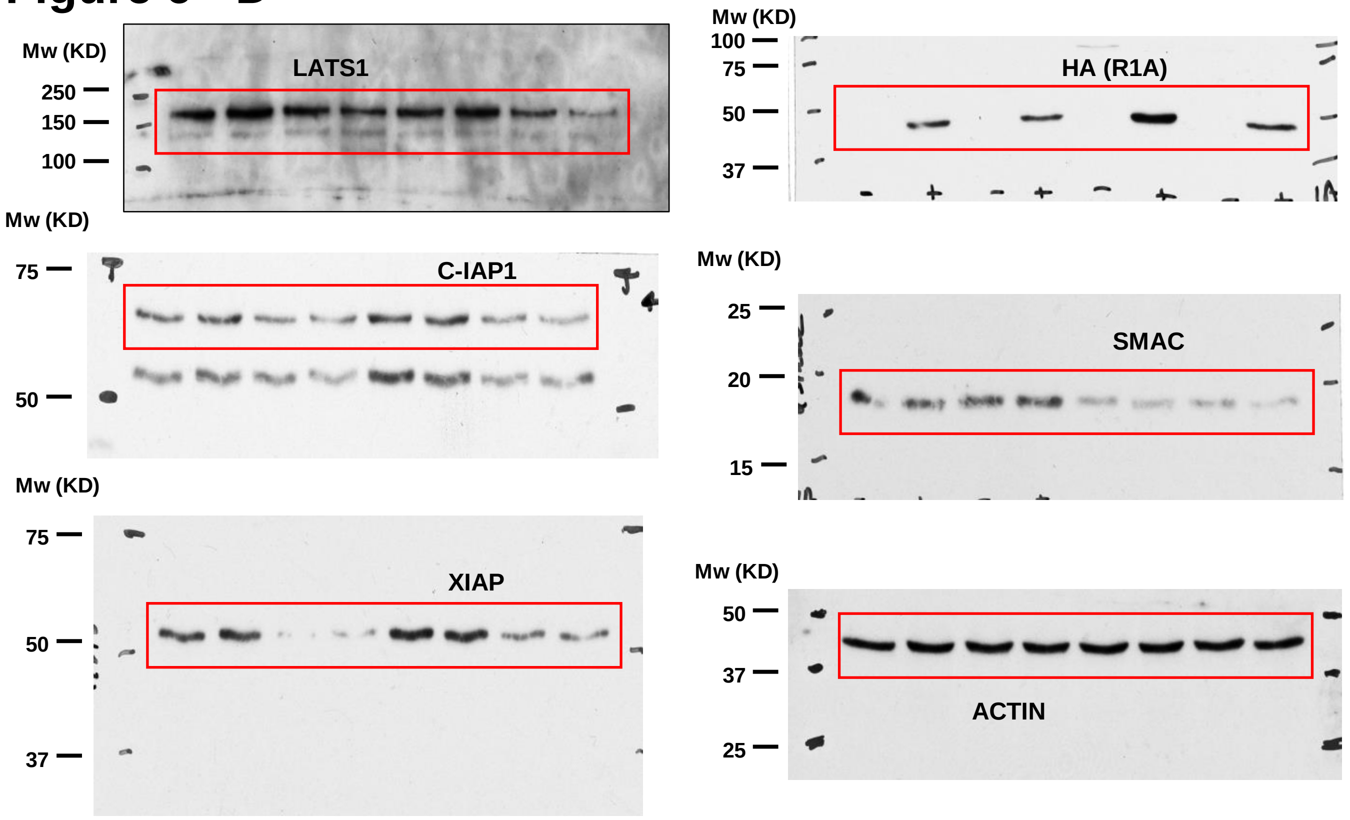

Figure 6 E

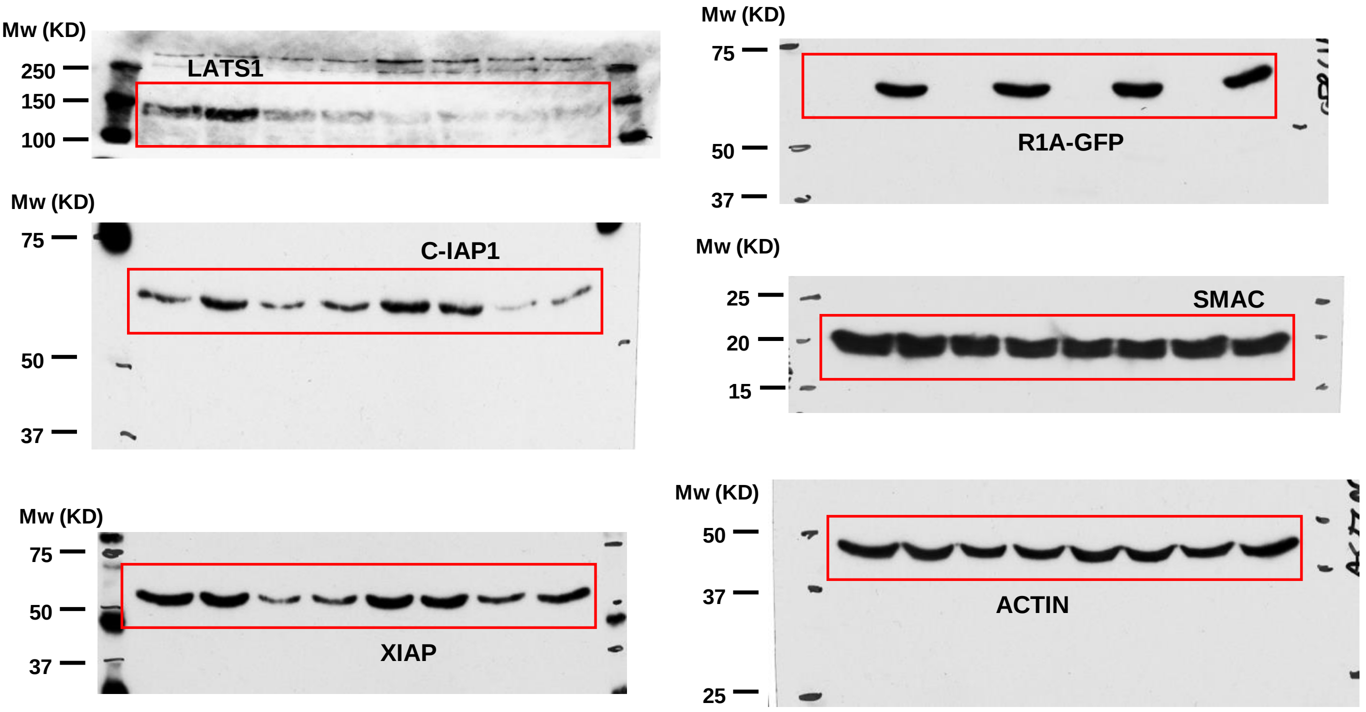

Figure 7 A

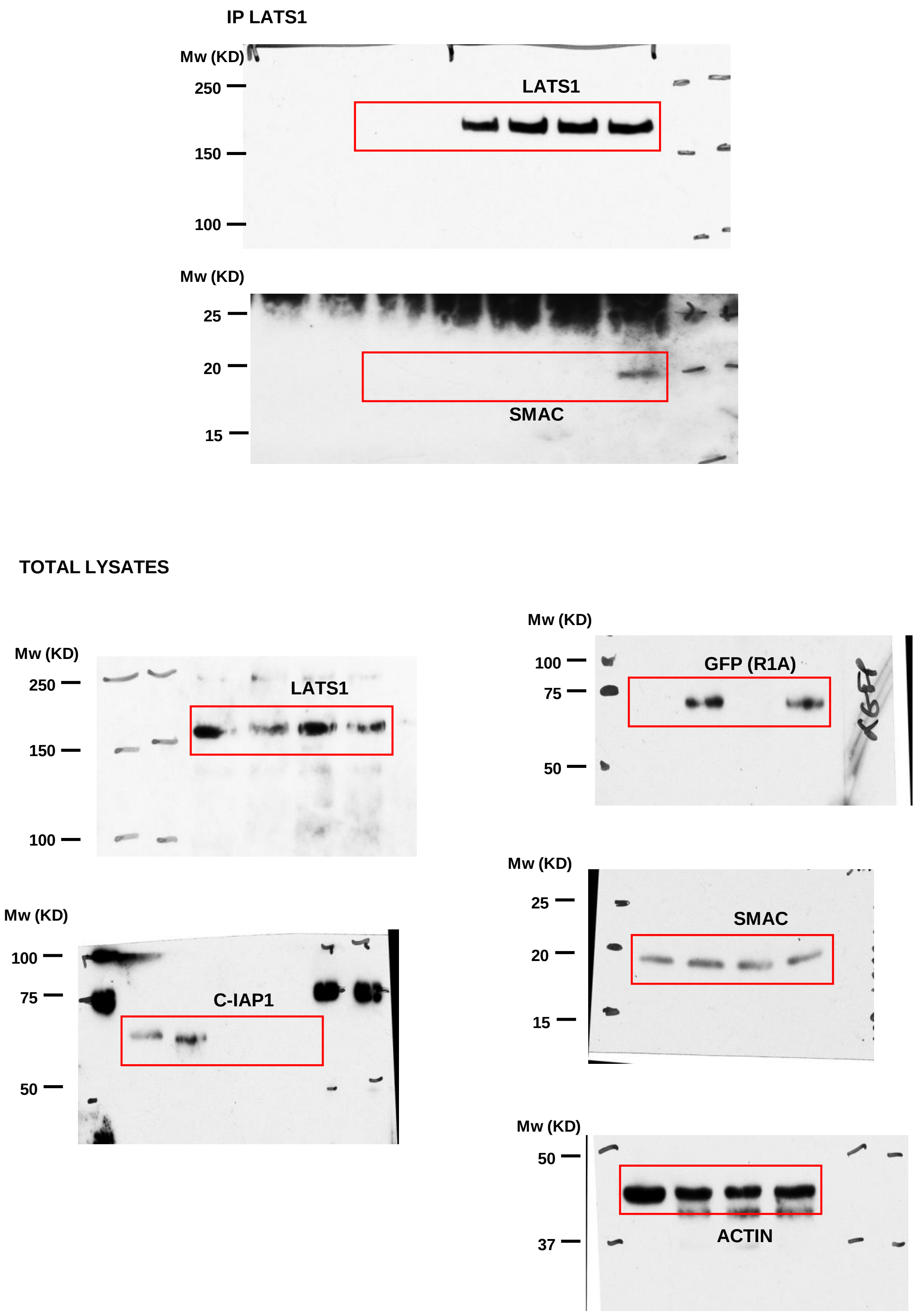

IP LATS1
Mw (KD)
250
150
100
LATS1
Mw (KD)
25
20
15
SMAC
TOTAL LYSATES
Mw (KD)
250
150
100
LATS1
Mw (KD)
100
75
50
GFP (R1A)
Mw (KD)
100
75
50
C-IAP1
Mw (KD)
25
20
15
SMAC
Mw (KD)
50
37
ACTIN

Figure 7 B

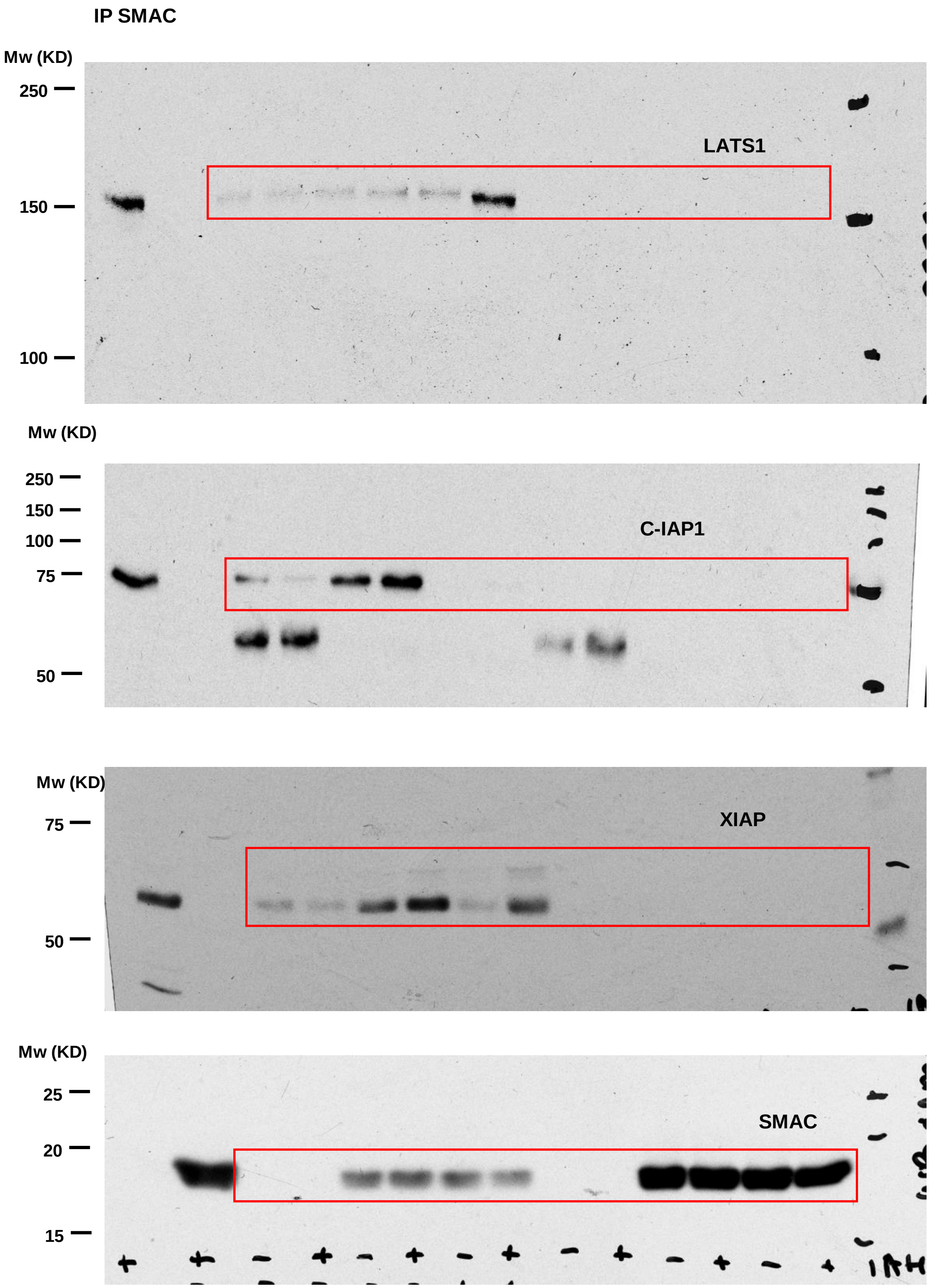
IP SMAC
Mw (KD)
250
150
100
LATS1
Mw (KD)
250
150
100
75
50
C-IAP1
Mw (KD)
75
50
XIAP
Mw (KD)
25
20
15
SMAC

Figure 7 B

TOTAL LYSATES

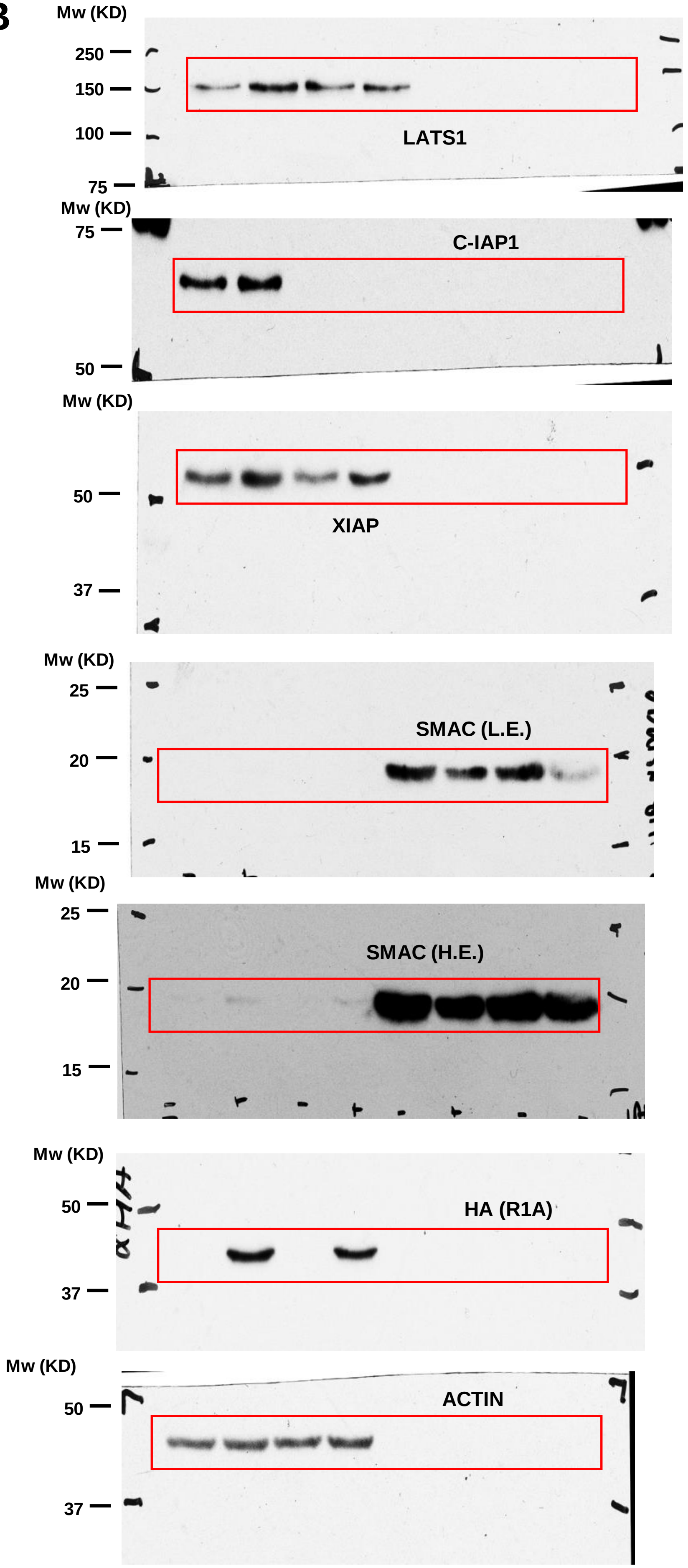

Figure 7 C

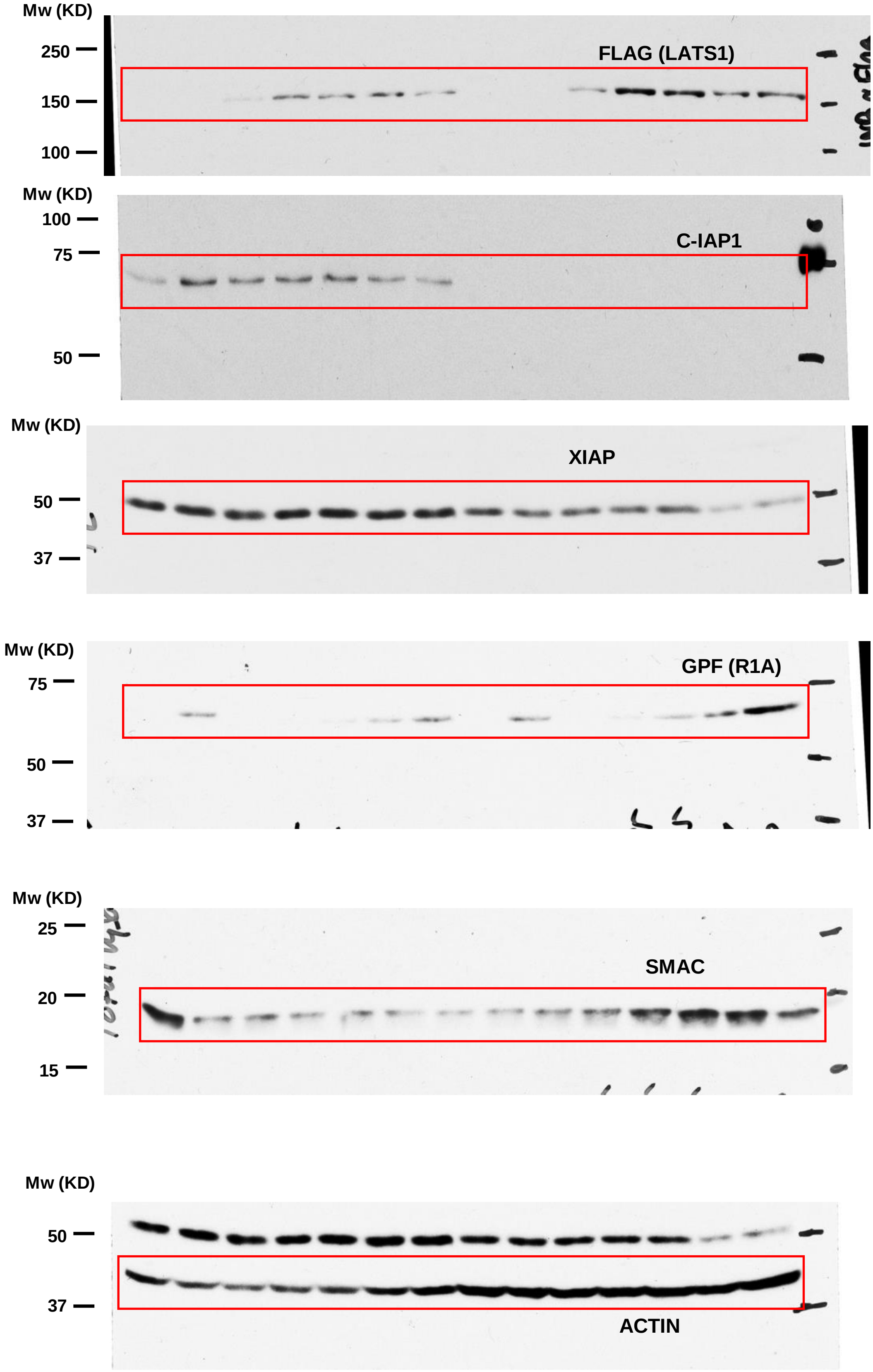
Mw (KD)
250
150
100
FLAG (LATS1)
Mw (KD)
100
75
50
C-IAP1
Mw (KD)
50
37
XIAP
Mw (KD)
75
50
37
GPF (R1A)
Mw (KD)
25
20
15
SMAC
Mw (KD)
50
37
ACTIN
