# Supplementary material for: Interaction of LATS1 with SMAC links the MST2/Hippo pathway with apoptosis in an IAP-dependent manner
Source: Cell Death Dis. 2022 Aug 8;13(8):692. doi: 10.1038/s41419-022-05147-3 (PMC9360443; doi:10.1038/s41419-022-05147-3)
Supplement: Supplementary file 3 — Supplementary figures [file 41419_2022_5147_MOESM3_ESM.pdf]

Supplementary Figure 1

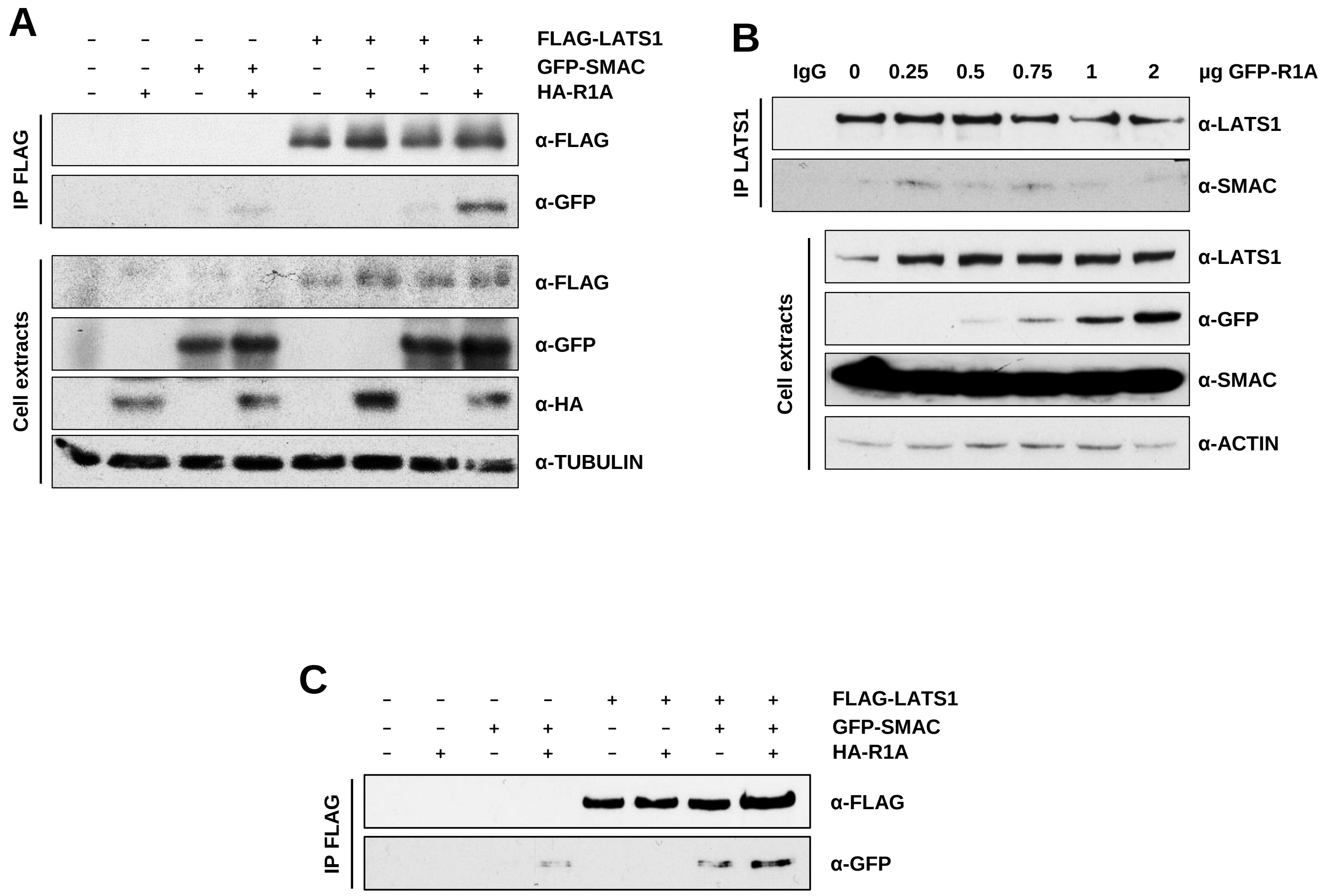

Supplementary Figure 1. A) Exogenous LATS1-SMAC interaction in A375 cells transfected with FLAG-LATS1 and GFP-SMAC assessed by co-immunoprecipitation assay upon RASSF1A re-expression followed by overnight serum deprivation. Levels of immunoprecipitated FLAG-LATS1 and co-immunoprecipitated GFP-SMAC are shown (upper panel). Total levels of transfected FLAG-LATS1, GFP-SMAC and HA-RASSF1A levels are shown (lower panel). Tubulin was used as loading control. **B)** LATS1-SMAC endogenous interaction in SK-MEL-239 melanoma cells upon increasing amounts of RASSF1A. Levels of immunoprecipitated LATS1 and co-immunoprecipitated SMAC are shown (upper panel). Normal IgG was used as negative control. Transfected GFP-RASSF1A and total levels of LATS1 and SMAC are shown (lower panel). ACTIN was used as loading control. **C)** Exogenous LATS1-SMAC interaction in SK-MEL-2 cells transfected with FLAG-LATS1 and GFP-SMAC assessed by co-immunoprecipitation assay upon RASSF1A re-expression followed by overnight serum deprivation. Levels of immunoprecipitated FLAG-LATS1 and co-immunoprecipitated GFP-SMAC are shown.

Supplementary Figure 2A

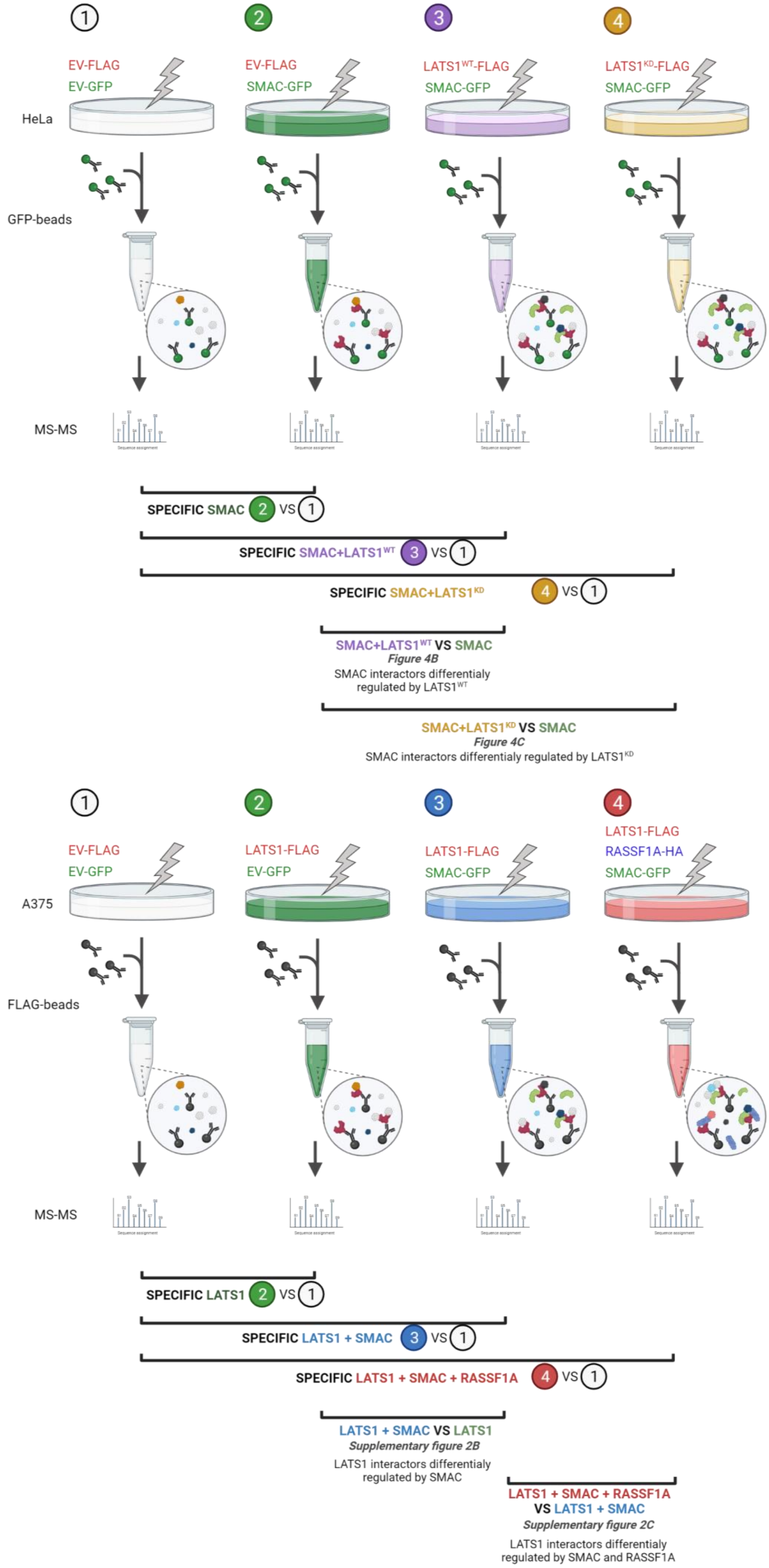

Supplementary Figure 2. A) Scheme depicting the AP-MS approaches used to study the dynamics of the LATS1-SMAC complex in HeLa (upper) and A375 (lower) cells.

Supplementary Figure 2B

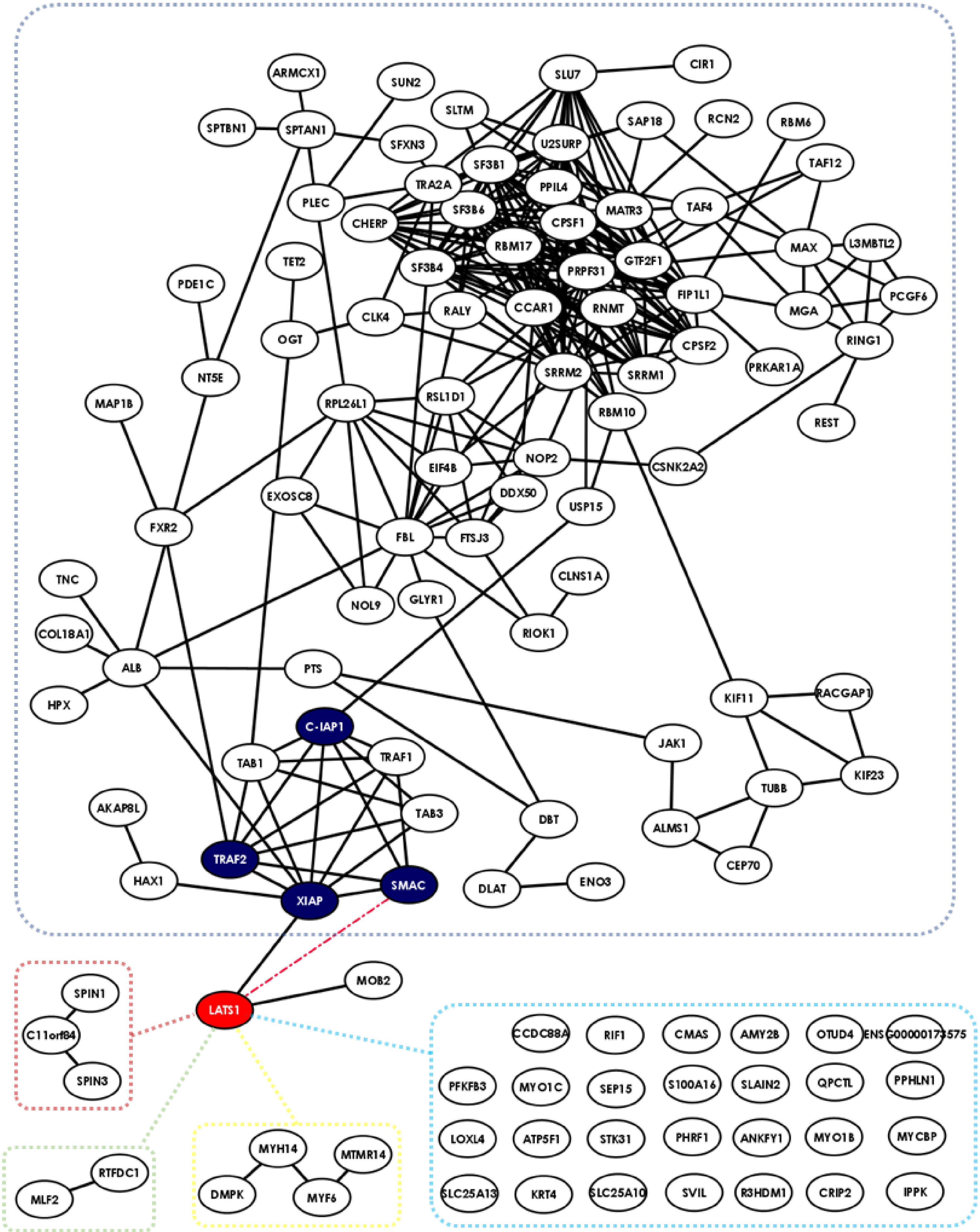

Supplementary Figure 2. B) Visualization of LATS1 interactors differentially regulated by SMAC after pathway reconstruction analysis (STRING database) in A375 cells. Red node represents the bait. Blue nodes represent proteins designated as the core components of the complex. Black edges represent reported interactions. Dash lines represent new interactions.

Supplementary Figure 2C

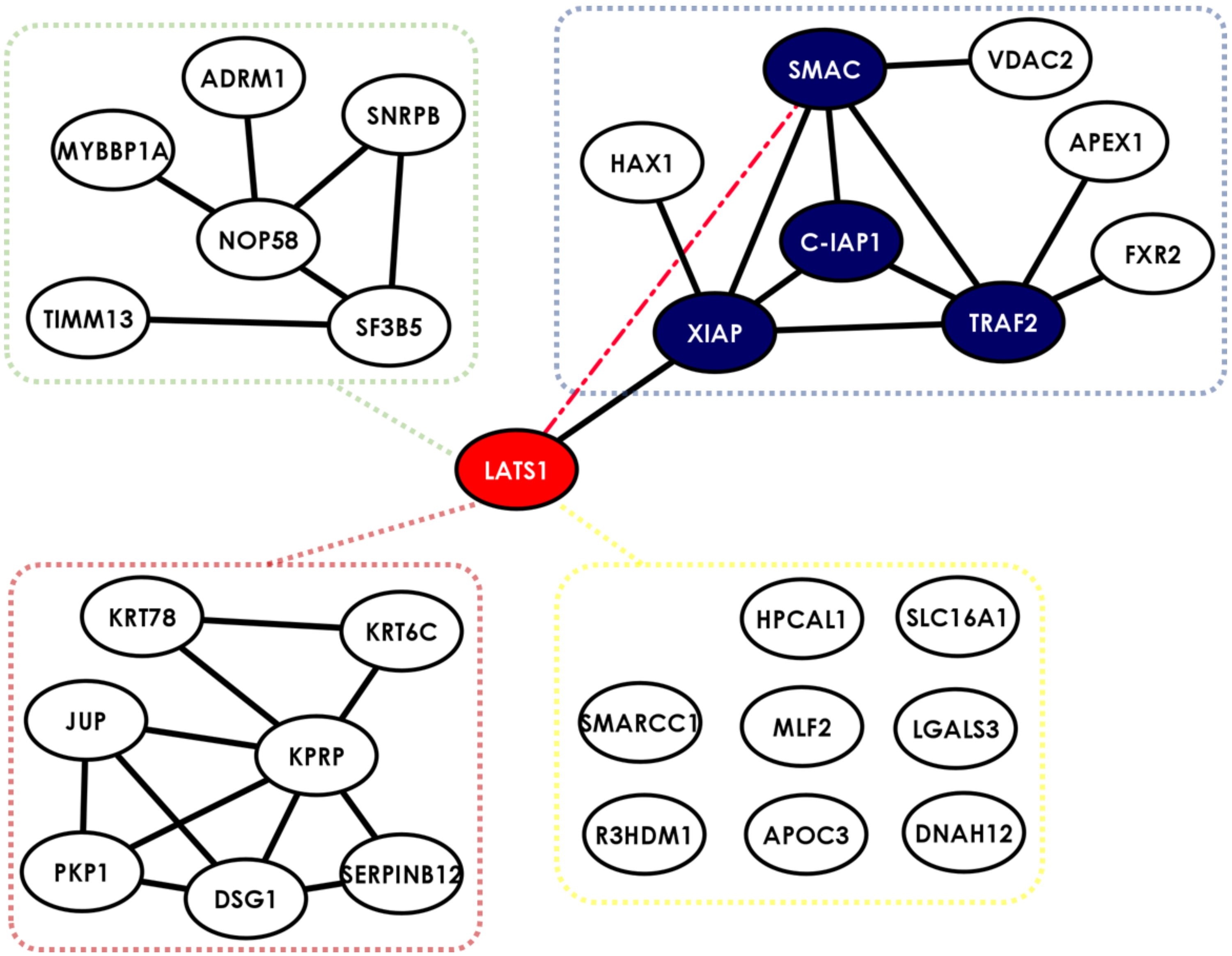

Supplementary Figure 2. C) Visualization of LATS1 interactors differentially regulated by SMAC and RASSF1A after pathway reconstruction analysis (STRING database) in A375 cells. Red node represents the bait. Blue nodes represent proteins designated as the core components of the complex. Black edges represent reported interactions. Dash lines represent new interactions.

Supplementary Figure 3

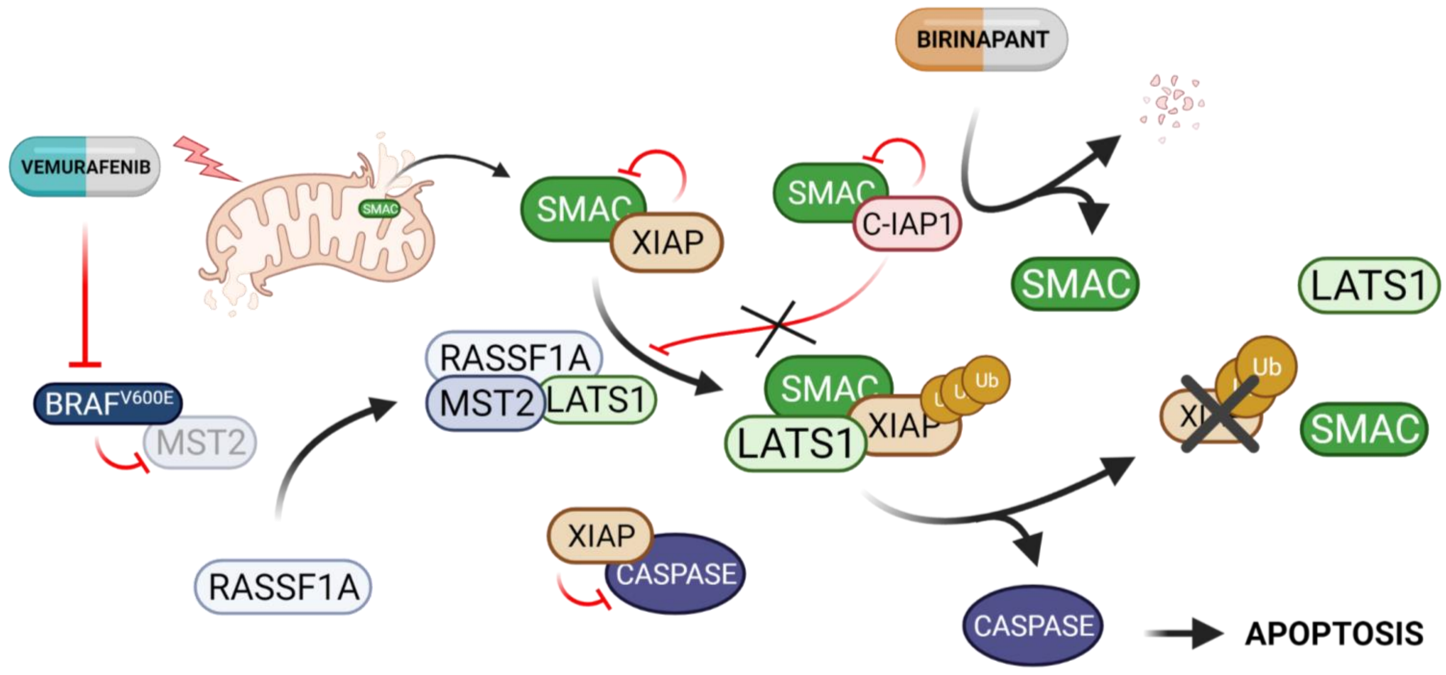

Supplementary Figure 3. Scheme depicting the model proposed for LATS1-SMAC mediated regulation of XIAP levels. The mitochondrial SMAC pro-apoptotic protein is released to the cytoplasm upon a proper stimuli. IAP members localized in the cytoplasm that inhibit both caspases and SMAC activity are counteracted by increasing levels of cytoplasmic SMAC and the formation of the LAST1-SMAC-XIAP complex which leads to XIAP ubiquitination and degradation promoting caspase activation. Birinapant enhances LATS1-SMAC complex formation by releasing C-IAP1 mediated inhibition over SMAC. RASSF1A promotes LATS1 activation through MST2 counteracting the inhibitory effect that BRAF^{V600E} exerts over the MST2 pathway and scaffolds LATS1-SMAC-XIAP complex formation. Vemurafenib induces LATS1-SMAC complex formation and XIAP degradation by inhibiting BRAF^{V600E}, promoting LATS1 activation and SMAC release from the mitochondria.
